# Altered effective connectivity of emotion perception and regulation networks during an emotional face perception task in adults with alcohol use disorder

**DOI:** 10.1007/s00429-025-02992-8

**Published:** 2025-08-14

**Authors:** Christopher J. Hammond, Liangsuo Ma, James M. Bjork, F. Gerard Moeller, Albert J. Arias

**Affiliations:** 1 Department of Psychiatry and Behavioral Sciences, Johns Hopkins University School of Medicine, 5500 Lombard Street, Baltimore, MD 21224 USA; 2 Institute for Drug and Alcohol Studies, Virginia Commonwealth University, Richmond, 23298 USA; 3 Department of Psychiatry, Virginia Commonwealth University, Richmond, 23298 USA; 4 Department of Pharmacology and Toxicology, Virginia Commonwealth University, Richmond, 23298 USA; 5 Department of Neurology, Virginia Commonwealth University, Richmond, 23298 USA; 6 Kenneth and Dianne Wright Center for Clinical and Translational Research, Virginia Commonwealth University (VCU), Richmond, VA 23298 USA; 7 Central Virginia Veterans Affairs Health Care System, Richmond, VA 23249 USA

**Keywords:** Alcohol use disorder, Dynamic causal modeling, Effective connectivity, Emotional face perception, Cumulative alcohol exposure, Depression

## Abstract

Impairments in emotional regulation and mood symptoms are interrelated and associated with alcohol use disorder (AUD) risk, but the underlying aberrant neural circuitry involved is poorly understood. In the present study, we examined alterations in effective (directional) connectivity (EC) during emotional face processing in individuals with and without AUD. We utilized functional MRI data from the Human Connectome Project obtained during an emotional face processing task in 70 participants with AUD and 70 controls (CON). Focusing on ventromedial prefrontal cortex (VMPFC), bilateral ventrolateral prefrontal cortex (VLPFC), amygdala (AMY), and fusiform gyrus (FG), and right (R) hypothalamus (HTN) nodes, we performed dynamic causal modeling analysis to test group-level differences in EC. Linear regressions characterized EC relationships with measures of cumulative alcohol exposure and depression and anxiety. Compared to CON participants, AUD participants had lower ECs from VMPFC → bilateral VLPFC, left (L)-VLPFC → L-VLPFC and VMPFC, R-VLPFC → L-FG, R-FG → HTN, and R-AMY → L-VLPFC; and greater ECs from VMPFC → VMPFC, L-VLPFC → R-VLPFC and bilateral FG, L-FG → R-AMY and HTN, R-AMY → VMPFC and L-FG, and L-AMY → HTN connectivities. In regression analyses, these cortical-to-cortical and cortical-to-subcortical ECs were associated with cumulative alcohol exposure. EC from R-VLPFC to L-FG was negatively associated with depression. Individuals with AUD have disrupted EC in cortical-to-cortical and cortical-to-subcortical circuits during emotional face processing in brain regions purported to govern emotion control, which may explain linkages between cumulative alcohol exposure and depression.

**Supplementary Information:**

The online version contains supplementary material available at 10.1007/s00429-025-02992-8.

## Introduction

Alcohol use disorder (AUD) is a complex heterogenous disorder that likely arises from various environmental exposures such as childhood adversity and risk-related phenotypes such as altered neural transmission in response to emotional stimuli (Sinha 2022). Recent studies of the phenomenology of AUD in terms of research domain criteria have attributed AUD to aberrations in three mechanistic neurobehavioral domains: incentive salience, negative emotionality, and executive/cognitive control (Kwako et al. 2016; Voon et al. 2020). Individual differences in the function, structure, and connectivity of neuronal circuits underlying these domains may drive development and maintenance of AUD and also could instantiate “biotypes” of AUD that identify candidates for certain AUD pharmacotherapies (Babor and Caetano 2006; Drysdale et al. 2017).

Of interest in this report is advancing the understanding of abnormalities in emotion perception and regulation circuity in AUD, particularly in relation to negative emotionality and social functioning (Koob 2017). Alterations in overt emotional perception and recognition and behavioral self-regulation during negative emotional provocation have been shown in individuals with AUD (Clark et al. 2007; Foisy et al. 2005; Kornreich et al. 2001; Le Berre 2019; Philippot et al. 1999) and mood disorders (Joormann and Stanton 2016; Krause et al. 2021) and may contribute to social functioning impairments in these populations (Imtiaz et al. 2018). Social impairments in AUD may stem from fundamental deficits in accurately perceiving social/emotional information, such as hostile attribution bias, wherein the individual perceives neutral social information as conveying hostile intent (Freeman et al. 2018). We posit that these socioemotional information processing (SIP) deficits in AUD stem from abnormal information flow in and out of brain structures thought to govern social and emotional information processing (Bjork et al. 2021).

Emotion-related circuit abnormalities have been observed in several functional magnetic resonance imaging (fMRI) studies. These have demonstrated altered neural activity and functional connectivity (FC) between regions of the emotion regulation and perception networks following exposure to visual emotional stimuli in people with AUD (Sinha 2022). For example, relative to controls, individuals with AUD had lower activation in prefrontal cortical (PFC) and limbic regions including the amygdala (AMY) and hippocampus (Heinz et al. 2007; Marinkovic et al. 2009; Muller-Oehring et al. 2013; Oberlin et al. 2012; Salloum et al. 2007) during negative emotional stimuli viewing. In addition, reduced FC between the AMY and medial and lateral PFC regions (dorsolateral PFC (DLPFC), ventromedial PFC (VMPFC), and orbitofrontal cortex) at rest and during emotion processing have been reported in AUD (Gorka et al. 2013; O’Daly et al. 2012; Seo et al. 2024; Sripada et al. 2011).

Reduced top-down PFC-AMY connectivity could result in compromised emotion regulation in this population (Ochsner et al. 2002; Quirk and Beer 2006). Similarly, poorer regulation of AMY by PFC when viewing emotional faces has been found in individuals with mood disorders, with altered AMY-PFC connectivity correlated with measures of anxiety, depression, and irritability in these populations (Etkin and Wager 2007; Kenwood et al. 2022; Kong et al. 2013; Porta-Casteras et al. 2020; Sheng et al. 2024; Stoddard et al. 2017).

Although emotion-related FC alterations have been observed in AUD, the directionality of disrupted information flow and magnitude of these relationships are poorly understood. Dynamic causal modeling (DCM) of fMRI time-series data can enable the measurement of effective (directional) connectivity (EC) (Friston et al. 2003, 2017). DCM of fMRI data models the excitatory and inhibitory effects from one brain region or network to another, while accounting for other influences (Snyder et al. 2021). Recent studies underscore that EC measured through DCM analysis do not show time-dependent variability related to diurnal variations in hemodynamic response (Frassle and Stephan 2022) and outperform standard FC for reliability of strong connectivities in both task-based and resting-state fMRI (Frassle and Stephan 2022). These strengths make DCM well-suited for hypothesis testing of competing top-down versus bottom-up circuit dysfunction hypotheses of psychopathology, improving our pathophysiological models of psychiatric disorders.

To date few studies have examined EC differences in AUD in the context of emotional stimuli. We found that AUD participants had greater ECs from insula and DLPFC to the anterior cingulate cortex (ACC) and ventral and dorsal striatum (VS/DS) and greater DS-to-DS self-connection EC during a number-guessing reward task than controls (Arias et al. 2021). Another study investigated EC differences during an emotional threat task, and found evidence of aberrant top-down threat processing with greater EC from the anteromedial thalamus to the insula in AUD (Radoman et al. 2024). Song and colleagues applied DCM to resting-state fMRI data in a sample of individuals with and without AUD, finding EC differences between default mode (DMN) and frontoparietal attention (FPN) networks that were bidirectional and correlated with alcohol-related problems (Song et al. 2021). No studies, however, have examined emotion-related EC during SIP in AUD.

In the present study, we sought to extend the current knowledge of AUD-related SIP deficits by examining EC during SIP in individuals with and without AUD using fMRI data collected during an emotional face-matching task (Hariri et al. 2002) from the Human Connectome Project (HCP) (Barch et al. 2013; Van Essen et al. 2013). Previous fMRI studies (e.g., Barch et al. 2013) have shown that this paradigm elicits a strong response in the AMY, hypothalamus (HTN), fusiform gyrus (FG), VMPFC, and ventrolateral PFC (VLPFC) in healthy individuals in response to negative emotional faces (Hariri et al. 2002). Based on existing models of neural circuit response to emotional face stimuli and previous fMRI/FC studies in AUD, we hypothesized that individuals with AUD would exhibit increased EC between the AMY and HTN, reflecting greater arousal and bottom-up emotional reactivity, and decreased EC between the VLPFC and AMY and VMPFC and AMY, reflecting reduced top-down emotion regulation. We also hypothesized that greater cumulative alcohol exposure would correlate with decreased ECs from the VLPFC and VMPFC to the AMY.

## Methods

### Data source, participants, general study procedures

Data used in this analysis were from the HCP-1200 Subjects Data Release. HCP study design, recruitment, data collection and processing procedures are detailed elsewhere (Barch et al. 2013; Glasser et al. 2013; Marcus et al. 2011; Van Essen et al. 2013; Van Essen et al. 2012). Use of HCP data was approved by the Virginia Commonwealth University Institutional Review Board.

**Fig. 1:**
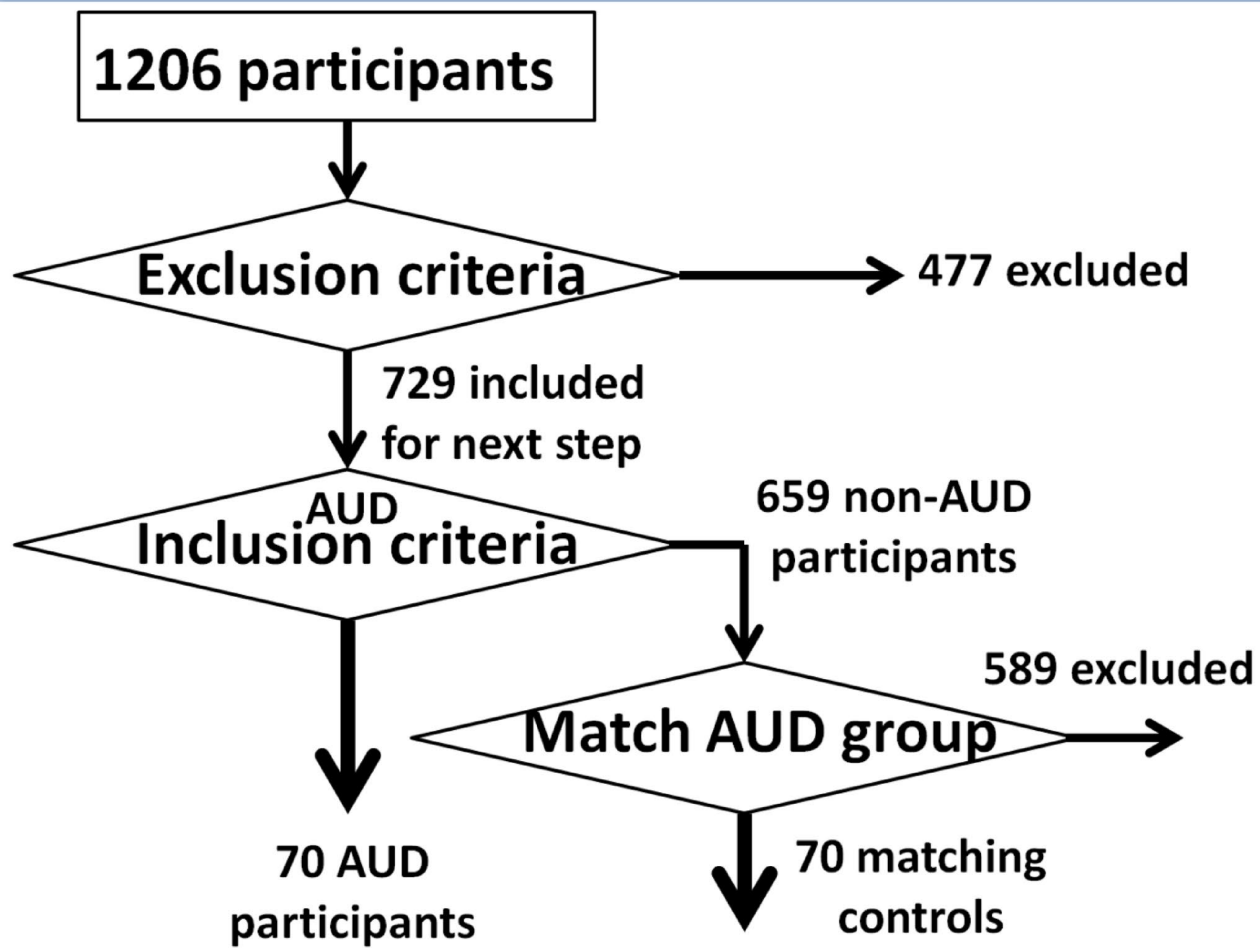
Participant selection procedure. See Table 1 and the materials and methods section for more detailed information about the matching between the alcohol use disorder (AUD) participants (*n* = 70) and the control participants (*n* = 70)

Participant selection criteria and procedures (Fig. 1) were modeled after Arias et al. (2021), and included some overlapping participants. When applied to the HCP 1206, these procedures identified 70 participants with AUD and 70 matched controls (CON). Nineteen exclusion criteria were used including handedness, non-alcohol substance use disorder (SUD) diagnosis, and history of regular/heavy drug use (see Table 1 for full list). Diagnostic ascertainment of AUD was obtained via the Semi-Structured Assessment for the Genetics of Alcoholism (SSAGA) (Bucholz et al. 1994). Similar to Arias et al. (2021), the control selection procedure was implemented by an in-house MATLAB code with different priority weights in matching demographics, cannabis use, and smoking between the candidate CON participants and the included AUD participants (see Supplementary Table S1).

**Table 1:** Participant exclusion criteria used in this study and the number of excluded participants

Exclusion criterion	Implementation of the exclusion criterion based on HCP measurements	Number of excluded participants
Handedness		
1. Not right handed	Handedness ≤ 0	117
Diagnosis on drug addiction		
2. Meeting cannabis dependence	SSAGA_Mj_Ab_Dep = one (1)	102 (51)
3. Meeting Fagerstrom nicotine dependence	SSAGA_FTND_Score = 4+	48 (13)
4. Meeting Fagerstrom tobacco dependence	SSAGA_HSI_Score = 4	0 (0)
Times of drug use		
5. Times used cocaine more than six times	SSAGA_Times_Used_Cocaine = 5	15 (6)
6. Times used hallucinogens more than six times	SSAGA_Times_Used_Hallucinogens = 5	1 (0)
7. Times used opiates more than six times	SSAGA_Times_Used_Opiates = 5	8 (5)
8. Times used sedatives more than six times	SSAGA_Times_Used_Sedatives = 5	1 (1)
9. Times used stimulants more than six times	SSAGA_Times_Used_Stimulants = 5	7 (5)
10. Times used marijuana more than 999 times	SSAGA_Mj_Times_Used = 5	22 (4)
Breath and urine test		
11. Alcohol breath test	Breathalyzer_Over_05 = “TRUE”	1
12. Positive urine test for THC	THC = “TRUE”	42
13. Positive urine test for cocaine	Cocaine = “TRUE”	0
14. Positive urine test for opiates	Opiates = “TRUE”	3
15. Positive urine test for amphetamines	Amphetamines = “TRUE”	4
16. Positive urine test for MethAmphetamine	MethAmphetamine = “TRUE”	1
17. Positive urine test for Oxycontin	Oxycontin = “TRUE”	1
Data availability		
18. Missing the measure of “total times used/smoked ANY TOBACCO in past 7 days”	Total_Any_Tobacco_7days = space (i.e., " “)	19
19. Emotional face task fMRI data are not complete	fMRI_Emo_PctCompl < 100%	85

The exclusion criteria were applied to all the 1206 HCP participants one by one (the number in the “Exclusion criterion” column represent the order of the exclusion criteria applied), resulting 729 participants remaining for subsequent consideration after excluding 477 participants who met at least one of the 19 exclusion criteria. Column 3 depicts the number of excluded subjects based upon each criteria from the total sample, with the number of excluded subjects with alcohol use disorder for each exclusion criteria shown in parentheses ()

### Emotional face perception task

The emotional face perception task used in HCP was adapted from (Hariri et al. 2002) and described in (e.g., Barch et al. 2013). In brief, participants are presented with alternating blocks of trials where they are shown images of emotional faces (angry or fearful expressions) or shapes, and instructed to selected which of the two faces/shapes on the bottom of the screen matches the face/shape at the top of the screen. Each block was 21 s, including a 3-second task cue (shape or face) followed by 6 trials of the same task (face or shape presenting for 2 s), which were separated by 1-second intervals.

### fMRI data acquisition and preprocessing

As described in (Barch et al. 2013), whole-brain gradient-echo, echo-planar fMRI imaging data was acquired with a 32-channel head coil on a modified 3-T Siemens Skyra scanner (Erlangen, Germany), with repetition time = 720 ms, echo time = 33.1 ms, flip angle = 52 degrees, bandwidth = 2290 Hz/pixel, in-plane field-of-view = 208 × 180 mm, 72 slices, 2.0 mm isotropic voxels, multi-band acceleration factor of 8. Two fMRI runs, with right-to-left and left-to-right phase encodings respectively, were acquired, preprocessed, and concatenated for DCM analysis.

For preprocessing: The HCP fMRI data used in this analysis underwent “minimal” preprocessing prior to release (Glasser et al. 2013). Preprocessing included gradient unwarping, motion correction, fieldmap-based EPI distortion correction, brain-boundary-based registration of EPI to structural T1-weighted scan, non-linear registration into standard MNI152 space, and grand-mean intensity normalization. Following Hillebrandt et al. (2014), we used Statistical Parametric Mapping 12 (SPM12) to spatially smooth the images with a 4-mm gaussian kernel. Head motion was then quantified using mean frame-wise displacement (FD) (Power et al. 2012) and compared between groups (Arias et al. 2021; Ma et al. 2021). FD results, presented in Table 2, show minimal motion and no group-wise differences.

**Table 2:** Demographic information (i.e., age, sex, race, and education), measures of alcohol cannabis use, and tobacco usage, for the included AUD (*n* = 70) and matching CON (*n* = 70) participants

Parameter	AUD (n = 70)	CON (n = 70)	Statistical results
Demographics			
Age (years) m, SD	29.0 ± 3.5 (22 to 36)	28.8 ± 3.5 (22 to 35)	t = 0.34, df = 138, *p* = 0.74
Sex	33 F, 37 M	38 F, 32 M	X^2^(1,140) = 0.71, *p* = 0.40
Race	59 White, 4 AA, 7 other	58 White, 7 AA, 5 other	X^2^(2,140) = 1.16, *p* = 0.56
Education (years) m, SD, (range)	15.6 ± 1.4 (11 to 17)	15.5 ± 1.5 (12 to 17)	t = 0.41, df = 138, *p* = 0.67
Cannabis use			
Lifetime cannabis dependence?	0	0	X^2^(1,140) = 0, *p* = 1
Tobacco usage			
Last 7 Days Tobacco Use	3.07 ± 10.70 (0 to 60)	2.99 ± 10.39 (0 to 56)	t = 0.04, df = 140, *p* = 0.96
Smoking history	never (n = 29) 1–19 times (n = 13) 20–99 times (n = 12) regular smoker (n = 16)	never (n = 33) 1–19 times (n = 12) 20–99 times (n = 11) regular smoker (n = 14)	X^2^(3,140) = 0.47, *p* = 0.92
Still smoking?	9 Yes, 61 No	8 Yes, 62 No	X^2^(1,140) = 0.07, *p* = 0.80
Fagerstrom FTND score	0 (n = 6) 1 (n = 4) 2 (n = 3) 3 (n = 3) No score (54)	0 (n = 7) 1 (n = 2) 2 (n = 3) 3 (n = 2) No score (56)	X^2^(4,140) = 0.98, *p* = 0.91
Anxiety and depression scores			
ASR DSM Anxiety Problems Raw Score	4.14 ± 3.16 (0 to 12)	4.07 ± 2.51 (0 to 12)	t = 0.15, df = 138, *p* = 0.88
ASR DSM Depressive Problems Raw Score	4.83 ± 4.02 (0 to 19)	3.86 ± 3.21 (0 to 14)	t = 1.58, df = 138, *p* = 0.12
Antisocial personality problems score			
ASR DSM Antisocial Personality Problems (scale 6) Raw Score	3.63 ± 3.12 (0 to 13)	3.04 ± 3.12 (0 to 15)	t = 1.11, df = 138, *p* = 0.27
Alcohol usage			
Total drinks in past 7 days (range)	8.21 ± 8.87 (0 to 47)	3.81 ± 4.33 (0 to 21)	t = 3.73, df = 138, *p* = 0.0003
Number of days drank alcohol in the past 7 days (range)	2.47 ± 1.71 (0 to 7)	2.40 ± 1.67 (0 to 7)	t = 0.25, df = 138, *p* = 0.807
Drinks per drinking day in past 12 months (range)	2.96 ± 1.60 (0 to 6)	2.00 ± 1.32 (0 to 5)	t = 3.87, df = 138, *p* = 0.0002
Frequency of any alcohol use in past 12 months (range) (smaller number represents larger frequency)	3.27 ± 1.36 (1 to 6)	4.54 ± 1.49 (1 to 6)	t = 5.27, df = 138, *p* < 0.0001
Drinks per day in heaviest 12-month period (range)	4.52 ± 1.47 (1 to 6)	3.29 ± 1.66 (1 to 6)	t = 4.64, df = 138, *p* < 0.0001
Frequency of any alcohol use, heaviest 12-month period (range) (smaller number represents larger frequency)	1.80 ± 1.04 (1 to 5)	3.76 ± 1.67 (1 to 6)	t = 8.34, df = 138, *p* < 0.0001
Averaged Z score for SSAGA measures of alcohol use (range)	0.39 ± 0.62 (‒1.02 to 2.28)	‒0.39 ± 0.60 (‒1.48 to 1.26)	t = 7.56, df = 138, *p* < 0.0001
Head motion during the fMRI scan			
Mean framewise displacement (mm)	0.16 ± 0.05 (0.08 to 0.32)	0.16 ± 0.06 (0.09 to 0.33)	t = 0.01, df = 138, *p* = 0.99
Behavioral performance during the task			
Accurate during emotional-face trails	Median: 1 IQR: 0.0333	Median: 1 IQR: 0.0333	Z = − 0.4571, ranksum =, *p* = 0.6476
Accurate during neutral-shape trials	Median: 0.9722 IQR: 0.0278	Median: 0.9722 IQR: 0.0278	Z = − 0.2058, ranksum = 4889*p* = 0.8370
Reaction time during emotional-face trails (ms)	Median: 772.75 IQR: 186.75	Median: 769 IQR: 133	Z = − 0.2271, ranksum = 4928, *p* = 0.9784
Reaction time during neutral-shape trails (ms)	Median: 748.625 IQR: 151	Median: 734.875 IQR: 134.74	Z = 0.0063, ranksum = 4937, *p* = 0.9950

Student-t and Chi-square tests were used to test the group difference in continuous and category variables respectively. Anxiety and depression scores, head motion during MRI scan, accuracy, and reaction times (RTs) during the task, for the included AUD (n = 70) and match CON (n = 70) participants. Variables with normally distribution are shown using mean ± standard deviation, and variables with non-normal distribution are shown using median and interquartile range (IQR). For these two kinds of variable, group differences were tested using Student-t and Wilcoxon rank sum tests respectively

*m* Mean, *SD* standard deviation, *AUD* alcohol use disorder, *CON* control

### Self-report assessment of alcohol and tobacco use

The SSAGA was used to measure recent and lifetime use of alcohol, tobacco and other drugs for HCP participants. Similar to previous studies (Arias et al. 2021; Ma et al. 2020; Orr et al. 2016), we quantified alcohol consumption using a composite measure created by averaging Z-scores from five SSAGA alcohol-related items: “Total drinks in past 7 days”, “Drinks per drinking day in past 12 months”, “Frequency of any alcohol use in past 12 months”, “Drinks per day in heaviest 12-month period”, and “Frequency of any alcohol use, heaviest 12-month period”. This composite measure reflects the cumulative effects of recent and/or past alcohol use (Orr et al. 2016). The SSAGA contains four tobacco-related items that can be similarly quantified (Orr et al. 2016), but only one (“Total times used/smoked any tobacco in past 7 days”) was completely available in the sample and used in our analyses.

### Anxiety and depression scores

Anxiety and depression were assessed in HCP using the Adult Self-report (ASR) questionnaire (Achenbach 2009), with raw subscale scores used in our analysis.

### SPM univariate analysis

Focusing on the contrast of face-matching minus shape-matching blocks, we conducted standard univariate analyses to test whether the two groups showed differences in contrast-elicited brain activation during the task. Following Ma et al. (2020), theses brain activations were also used to functionally-localize the nodes to be used for DCM. Anatomical labels for regions of activation were determined using the Anatomical Automatic Labeling (AAL3) atlas (Rolls et al. 2020) and hypothalamus atlases (Baroncini et al. 2012; Lemaire et al. 2013). For all second-level SPM univariate analyses, the cluster-defining threshold was *p* =.001; statistical significance was defined as family-wise error (FWE) corrected cluster probability *p* <.05 (two-tailed).

### Dynamic causal modeling

FMRI-based DCM is a biophysical model of how the neuronal connectivity generates the observed fMRI signal (Friston et al. 2003, 2019). DCM has been described in detail elsewhere (Friston et al. 2003; Ma et al. 2012, 2014, 2020). In brief, DCM is a dynamical system of bilinear differential state equations with coefficients (in units of Hz). A node in the model that receives driving input is the brain region which first experiences a task-elicited change in neuronal activity. This node then influences other nodes. The endogenous connectivity measures the EC strengths between nodes, regardless of the moment-to-moment switching on and off of inputs. Experimental conditions can modulate the endogenous connectivities. The parameters of the modulation effects quantify increased or decreased connectivity strength compared to the endogenous ECs.

The DCM analysis was conducted by following (Ma et al. 2020). A parametric regressor called “All-visual-stimuli” (reflecting common features of the emotional-face and neutral-shape stimuli) was used as a single driving input to the DCM, and another parametric regressor called emotional-face minus neutral-shape (face–shape), reflecting the special effect of face over shape, was used as a single modulator of EC. The changes of ECs corresponding to the face–shape modulator (relative to the endogenous ECs) are termed modulatory changes. By definition, a modulatory change reflects the change in EC corresponding to the emotional-face trials minus the change in EC corresponding to the neutral-shape trials. Hereafter, all ECs refer to the modulatory changes as defined here.

The DCM nodes (regions of interest) were determined in two steps. In Step 1, before looking at the brain activation results, some candidate nodes were selected based on neuroimaging studies investigating reaction to unpleasant events (Carretie et al. 2009), stress reactivity (Arnsten 2009), face perception (Haxby et al. 2000), and alcohol and drug addiction (Koob and Volkow 2016), and relevant fMRI studies reviewed in the introduction. These a priori selected candidate DCM nodes were: VMPFC, bilateral VLPFC, bilateral AMY, bilateral FG, and HTN. In Step 2, we further evaluated whether a candidate region should be selected as a final region based on the actual brain activation results observed in this study. Specifically, if the putative region showed activation unique to the participants, it was chosen. The location of the DCM node (a sphere) within the final region was then determined, with the center of the sphere placed at the point of maximum significance in brain activation within the selected region. Eight brain regions, i.e., VMPFC, left and right VLPFC, AMY, and FG, and right HTN, were selected as the final DCM nodes.

The spatial extent of each brain structure for DCM node placement was determined using the AAL3 atlas and hypothalamus atlases (Baroncini et al. 2012; Lemaire et al. 2013). In AAL3, the anatomical descriptions corresponding to VMPFC, VLPFC, AMY, and FG regions were superior frontal gyrus (medial orbital), inferior frontal gyrus (opercular part, triangular part, and pars orbitalis), AMY, and FG respectively. Each DCM node was defined as a 5-mm-radius sphere centered at the local maximum (z value) within each brain region in the activation found in this study. For each participant, the same nodes were initialized but were slightly re-positioned based on that participant’s local activation maxima (see Supplementary Table S2 for the MNI coordinates of the original DCM nodes and the re-positioned DCM nodes). With the motion parameters (x, y, and z translations and rotations) as the covariates, the principal eigenvariate (Stephan et al. 2010) was extracted from each DCM node and used as a summary of the time series for that DCM node.

The group-level analyses for the EC modulatory changes were conducted using the DCM Parametric Empirical Bayesian (PEB) approach (Friston et al. 2016), as implemented in SPM12 (Revision 7771), and outlined below: (I) testing each modulatory EC against zero in the CON group; (II) testing the group differences in each modulatory EC between the two groups (AUD-CON); and (III) testing the linear relationship between each modulatory EC and other measures of interest (e.g., alcohol usage). Specifically, the function “spm_dcm_peb_fit” was used to iteratively re-estimate all the single subject DCMs, each time setting the priors on the connectivity parameters to the group average (as determined using spm_dcm_peb). The function “spm_dcm_peb” was used to perform a group-level analysis, which specified a hierarchical model with DCMs at the subject level and a GLM at the group level.

The advantage of the linear regression analysis within the PEB framework is that the covariance among all DCM parameters in the model is automatically taken into consideration. PEB uses Bayesian posterior inference (Friston and Penny 2003) in which posterior probability (Bayesian-PP) is used as an indicator of the confidence in whether a modulatory EC in a group is different from zero (or different compared to another group) or the confidence in the degree of the linear relationship between variables. The Bayesian-PP (0 ≤ Bayesian-PP ≤ 1) is the conditional probability that is computed by PEB using Bayes rule after the available information (the likelihood function and the prior probability density of the models parameters) is taken into account (Friston and Penny 2003). The higher the Bayesian-PP, the greater the confidence. In this paper, an EC finding was considered reliable if Bayesian-PP > 0.95 (corresponding to a Bayes-factor of 20). In Bayesian statistics, the concept of “significance” does not apply. Instead, we deal with the probability of a particular effect or model. As recommended by the DCM authors in the SPM Archives, the discussion of DCM results should emphasize the most probable effects—typically those with probabilities exceeding 90% or 95%. Therefore, by selecting Bayesian-PP > 0.95, we ensured that our analysis focused on the most probable effects. Bayesian posterior inference during the PEB analyses eschews the multiple-comparison problem because of the lack of false positives (Friston et al. 2003; Friston and Penny 2003; Van Overwalle et al. 2019). Specifically, the PP of an EC, given the data, is the same, irrespective of whether all the ECs in the same DCM were analyzed or not (Friston and Penny 2003). This is implemented by using independent prior distributions for different ECs (Friston and Penny 2003).

Supplemental DCM analyses were also conducted to test for sex differences and group-by-sex interaction effects.

## Results

### AUD vs. CON group comparisons of self-report and behavioral measures

As shown in Table 2, the AUD group had significantly higher alcohol consumption compared to CON, but there were no group differences in demographics, tobacco and cannabis use or dependence rates, depression and anxiety scores, antisocial personality problems score, and head motion and behavioral performance during the fMRI scans.

### Univariate analysis of contrast-elicited BOLD response in AUD and CON groups

**Fig. 2:**
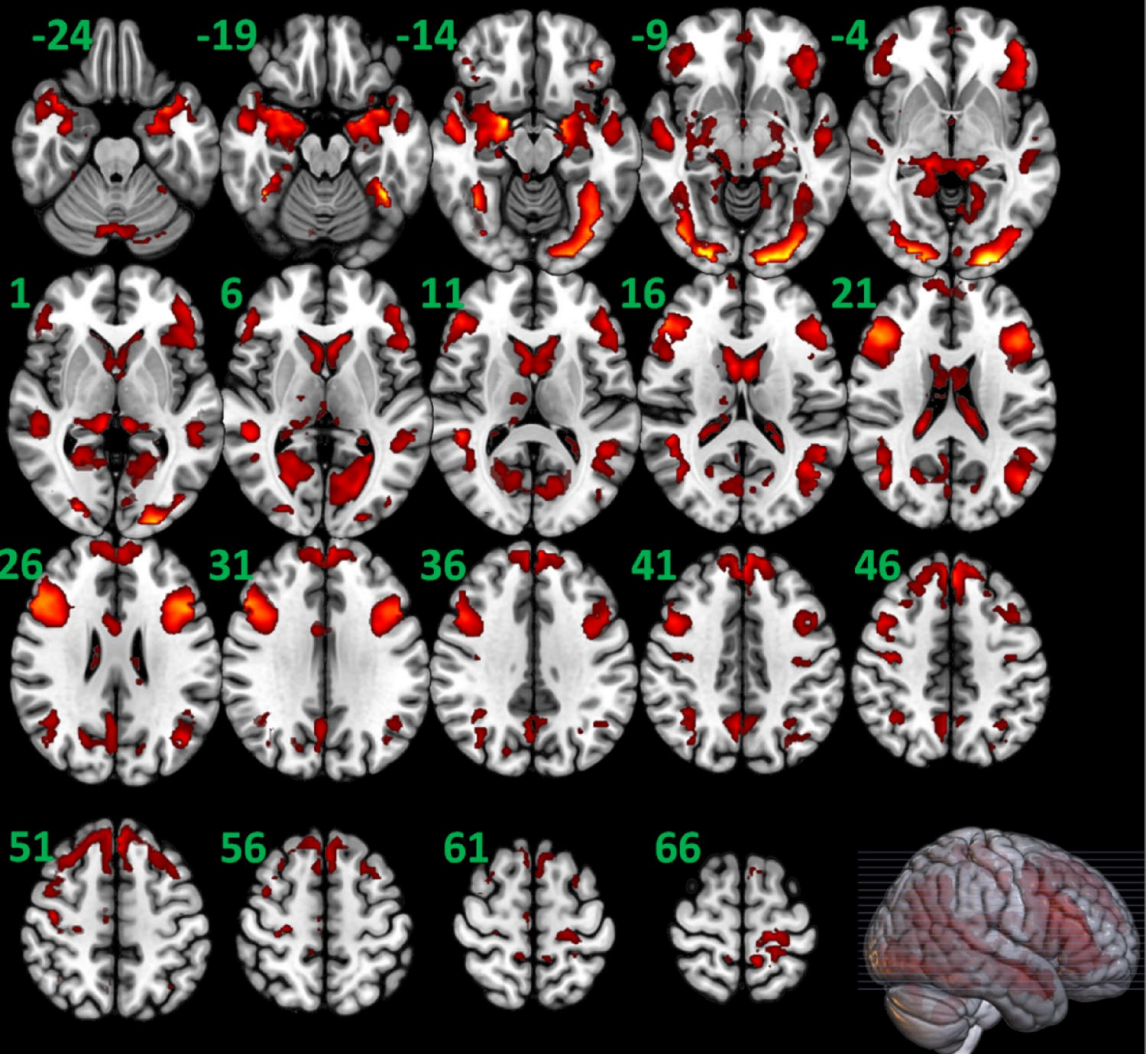
The brain activation clusters detected by SPM12 second-level random effects 1-sample t test analysis for the contrast of emotional-face minus neutral-shape when the two groups were combined (*n* = 140), with cluster-defining threshold *p* =.001 and cluster level *p* <.05 (FWE corrected, two-tail). The clusters are overlaid in color on axial slices of the Montreal Neurological Institute brain template image in gray. The number near the top/upper-left corner of each slice indicates slice location (mm) of the MNI z coordinate. The reader’s left side of each slice is the subjects’ left brain hemisphere

SPM two-sample t-test analyses showed no statistically significant differences (FWE corrected cluster *p* >.100, two-tail) in simple linear contrasts of task contrast-elicited activation (i.e., emotional-face– neutral-shape) between the two groups, thus supporting our plan to localize nodes for DCM based on the second-level map of the combined groups. When the two groups were combined (*n* = 140), the group level analyses found several statistically significant (FWE corrected two-tail cluster level *p* <.001) activation clusters (see Fig. 2 and Supplementary Table S3). Cluster results were used to guide selection and seed location of final nodes from the a priori candidates.

### DCM PEB analysis

**Fig. 3:**
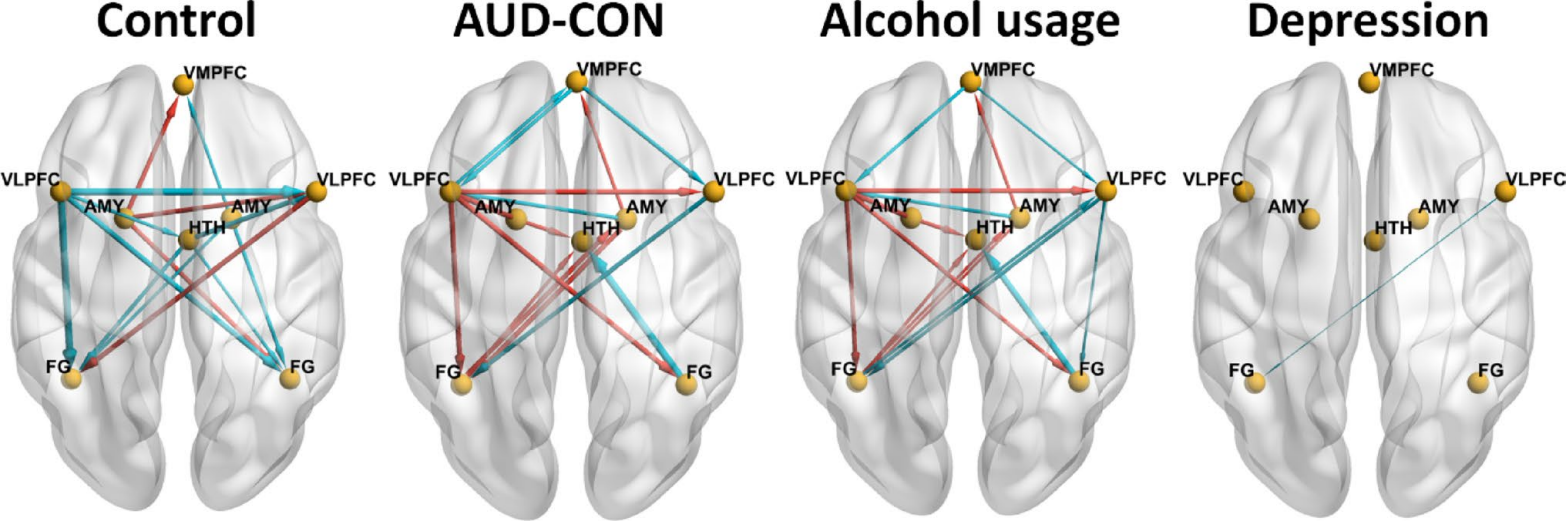
The main results of the analyses of group comparison in effective connectivities (ECs) and the analyses of the linear regression between ECs and cumulative alcohol usage and depression, visualized with the BrainNet Viewer (http://www.nitrc.org/projects/bnv/) (Xia et al. 2013). For the group comparisons, lines with arrows representing if ECs were different from zero in the Control group (*n* = 70, posterior probability [PP] > 0.95, left panel) or the group difference (AUD [*n* = 70] minus CON [*n* = 70]), PP > 0.95, the second panel from left) in ECs. A red line denotes that an EC was greater than zero in the Control group (left panel) or greater in the AUD group than the CON group (the second panel from left); and a blue line denotes that an EC was smaller than zero in the Control group (left panel) or smaller in the Alcohol group than the Control group (the second panel from left). Line width is proportional to the EC strength. For the linear regression analyses (across all participants, *n* = 140), lines with arrows representing if ECs showed linear relationship with the cumulative alcohol usage (PP > 0.95, the third panel from left) or the depression (PP = 0.89, the right panel). A red line denotes that an EC showed positive linear relationship with the cumulative alcohol usage (the third panel from left); and a blue line denotes that an EC showed negative linear relationship with the cumulative alcohol usage (third panels from left) or the depression (the right panels). Line width is proportional to the linear regression coefficient. *AUD* alcohol use disorder, *CON* control, *VMPFC* ventrolateral prefrontal cortex, *VLPFC* ventrolateral prefrontal cortex, *AMY* amygdala, *FG* fusiform gyrus, *HTH* hypothalamus

*AUD vs. CON group comparisons*: The group differences in each EC between AUD and CON participants and corresponding Bayesian-PP, together with the ECs and corresponding Bayesian-PP found in the CON group, are shown in Table 3. Since the test-retest reliability of the ECs with larger magnitude (i.e., absolute value) is higher (Frassle and Stephan 2022), we focus our results presentation and discussion on larger magnitude connectivities (|EC|≥0.3 Hz, PP = 1) for the CON group, followed by AUD-CON between-group differences. We did not specifically focus on larger ECs in the AUD group due to our analysis design. First, we measured ECs in the CON group as a direct measure. Then, we assessed how the AUD group differed from the CON group, making the ECs in the AUD group an indirect measure relative to the CON group. This approach ensures that our analysis captures between-group differences in ECs which are meaningful in healthy individuals. All EC analyses are shown in Fig. 3 (see Fig. 3A and B for EC group and AUD-CON comparison analysis). In the CON group analysis, ECs from left VLPFC → left VLPFC, right VLPFC → left FG, left FG → left FG, and left AMY → VMPFC, right VLPFC, and right FG elicited by face-blocks as driving input were positive, and ECs from left VLPFC → right VLPFC and bilateral FG, right VLPFC → right VLPFC, right AMY → VMPFC and bilateral FG, and HTN → right VLPFC and bilateral FG were negative (all PPs were 1). In the AUD vs. CON group analysis, the AUD group had lower ECs from VMPFC → bilateral VLPFC, left VLPFC → left VLPFC and VMPFC, right VLPFC → left FG, right FG → HTN, and right AMY → left VLPFC; and greater EC from VMPFC → VMPFC, left VLPFC → right VLPFC and bilateral FG, left FG → right AMY and HTN, right AMY ◊VMPFC and left FG, and left AMY → HTN compared to the CON group (all PPs > 0.95).

**Table 3:** The results of the group level DCM PEB analyses

	EC in CON participants (*n* = 70)		Group difference: AUD (*n* = 70) minus CON (*n* = 70) during		Linear regression between EC and cumulative alcohol use in combining AUD and CON participants (*n* = 140)		Linear regression between EC and anxiety score in combining AUD and CON participants (*n* = 140)		Linear regression between EC and depression score in combining AUD and CON participants (*n* = 140)	
Connectivity	EC (Hz)	PP	EC (Hz)	PP	Beta	PP	Beta	PP	Beta	PP
VMPFC → VMPFC	0.0000	0	0.6159	1	0.2465	1	0.0000	0	0.0000	0
VMPFC → L VLPFC	0.2028	1	− 0.3510	1	− 0.1518	1	0.0000	0	0.0000	0
VMPFC → R VLPFC	0.1117	1	− 0.3825	1	− 0.1218	1	0.0000	0	0.0000	0
VMPFC → L AMY	0.1589	1	0.0000	0	0.0000	0	0.0000	0	0.0000	0
VMPFC → R AMY	0.0568	0.8055	− 0.1856	1	− 0.0907	1	0.0000	0	0.0000	0
VMPFC → L FG	0.2434	1	− 0.1689	1	0.0000	0	0.0000	0	0.0000	0
VMPFC → R FG	0.1181	1	0.0000	0	0.0000	0	0.0000	0	0.0000	0
VMPFC → HTH	0.0000	0	0.0000	0	0.0000	0	0.0000	0	0.0000	0
L VLPFC → VMPFC	0.0000	0	− 0.3156	1	− 0.1003	0.8681	0.0000	0	0.0000	0
L VLPFC → L VLPFC	0.9375	1	− 0.4980	1	− 0.1597	0.8239	0.0000	0	0.0000	0
L VLPFC → R VLPFC	− 0.6464	1	0.4553	1	0.2255	1	0.0000	0	0.0000	0
L VLPFC → L AMY	− 0.2793	1	0.0000	0	0.0000	0	0.0000	0	0.0000	0
L VLPFC → R AMY	− 0.2506	1	0.0000	0	0.0000	0	0.0000	0	0.0000	0
L VLPFC → L FG	− 0.6946	1	0.4779	1	0.2050	1	0.0000	0	0.0129	0.6358
L VLPFC → R FG	− 0.5150	1	0.4890	1	0.1981	1	0.0000	0	0.0000	0
L VLPFC → HTH	− 0.3324	1	0.0000	0	0.0000	0	0.0000	0	0.0000	0
R VLPFC → VMPFC	0.0000	0	0.0000	0	0.0000	0	0.0000	0	0.0000	0
R VLPFC → L VLPFC	0.1148	1	0.0000	0	0.0000	0	0.0000	0	0.0000	0
R VLPFC → R VLPFC	− 0.4543	1	0.0000	0	0.0000	0	0.0000	0	0.0000	0
R VLPFC → L AMY	0.1303	1	0.1575	1	0.0000	0	0.0000	0	0.0000	0
R VLPFC → R AMY	0.1827	1	0.0000	0	0.0000	0	0.0000	0	0.0000	0
R VLPFC → L FG	0.4897	1	− 0.4211	1	− 0.1693	1	0.0000	0	− 0.0205	0.8909
R VLPFC → R FG	0.2411	1	− 0.2429	1	− 0.1288	1	0.0000	0	0.0000	0
R VLPFC → HTH	− 0.1416	1	0.1719	1	0.0000	0	0.0000	0	0.0000	0
L AMY → VMPFC	0.4364	1	0.0000	0	0.0000	0	0.0000	0	0.0000	0
L AMY → L VLPFC	0.0992	0.9875	0.4874	1	0.2341	1	0.0000	0	0.0000	0
L AMY → R VLPFC	0.4580	1	0.0000	0	0.0000	0	0.0000	0	0.0000	0
L AMY → L AMY	0.2796	1	0.0000	0	− 0.1286	0.8458	0.0000	0	0.0000	0
L AMY → R AMY	0.2478	1	0.0000	0	0.0000	0	0.0000	0	0.0000	0
L AMY → L FG	0.2806	1	0.0000	0	0.0000	0	0.0000	0	0.0000	0
L AMY → R FG	0.4434	1	0.0000	0	0.0000	0	0.0000	0	0.0000	0
L AMY → HTH	0.2141	1	0.3661	1	0.1825	1	0.0000	0	0.0000	0
R AMY → VMPFC	− 0.3114	1	0.3285	1	0.1547	1	0.0000	0	0.0000	0
R AMY → L VLPFC	0.2369	1	− 0.3577	1	− 0.1644	1	0.0000	0	0.0000	0
R AMY → R VLPFC	− 0.0910	0.9346	0.2464	1	0.0000	0	0.0000	0	0.0000	0
R AMY → L AMY	0.1044	1	0.2204	1	0.0000	0	0.0000	0	0.0000	0
R AMY → R AMY	0.2456	1	0.0000	0	0.0000	0	0.0000	0	0.0000	0
R AMY → L FG	− 0.3199	1	0.4503	1	0.1242	1	0.0000	0	0.0000	0
R AMY → R FG	− 0.3670	1	0.1905	1	0.0000	0	0.0000	0	0.0000	0
R AMY → HTH	− 0.1925	1	0.0000	0	0.0000	0	0.0000	0	0.0000	0
L FG → VMPFC	0.2363	1	0.2053	1	0.0000	0	0.0000	0	0.0000	0
L FG → L VLPFC	− 0.1890	1	0.1168	0.8509	0.0000	0	0.0000	0	0.0000	0
L FG → R VLPFC	0.2071	1	− 0.2071	1	− 0.1381	1	0.0000	0	0.0000	0
L FG → L AMY	0.1541	1	0.0000	0	0.0000	0	0.0000	0	0.0000	0
L FG → R AMY	0.0000	0	0.4481	1	0.1457	1	0.0000	0	0.0000	0
L FG → L FG	0.4013	1	− 0.2521	0.9849	0.0000	0	0.0000	0	0.0000	0
L FG → R FG	0.0381	0.5499	− 0.1218	1	0.0000	0	0.0000	0	0.0000	0
L FG → HTH	0.0000	0	0.3126	1	0.1538	1	0.0000	0	0.0000	0
R FG → VMPFC	− 0.1838	1	0.0000	0	0.0000	0	0.0000	0	0.0000	0
R FG → L VLPFC	0.1988	1	− 0.0799	0.653	0.0000	0	0.0000	0	0.0000	0
R FG → R VLPFC	0.0000	0	0.0000	0	0.0000	0	0.0000	0	0.0000	0
R FG → L AMY	0.0000	0	− 0.1716	1	0.0000	0	0.0000	0	0.0000	0
R FG → R AMY	− 0.2093	1	− 0.2527	1	0.0000	0	0.0000	0	0.0000	0
R FG → L FG	0.2873	1	− 0.1603	1	0.0000	0	0.0000	0	0.0000	0
R FG → R FG	− 0.2379	1	0.0000	0	0.0000	0	0.0000	0	0.0000	0
R FG → HTH	0.2364	1	− 0.6868	1	− 0.2913	1	0.0000	0	0.0000	0
HTH → VMPFC	− 0.2539	1	0.1964	1	0.0745	0.79	0.0000	0	0.0000	0
HTH → L VLPFC	− 0.2924	1	0.0000	0	0.0000	0	0.0000	0	0.0000	0
HTH → R VLPFC	− 0.4795	1	0.0000	0	0.0000	0	0.0000	0	0.0000	0
HTH → L AMY	− 0.1926	1	0.0000	0	0.0000	0	0.0000	0	0.0000	0
HTH → R AMY	0.0000	0	0.0000	0	0.0000	0	0.0000	0	0.0000	0
HTH → L FG	− 0.3809	1	− 0.1678	1	− 0.0798	0.8408	0.0000	0	0.0000	0
HTH → R FG	− 0.3109	1	− 0.1312	0.983	0.0000	0	0.0000	0	0.0000	0
HTH → HTH	− 0.2755	1	0.0000	0	0.0000	0	0.0000	0	0.0000	0

For each node-to-node connection in the each of the group level PEB analyses, an EC magnitude (absolute value in Hz) and bayesian posterior probabilities (PP) between 0 and 1 (0 ≤ PP ≤ 1) is shown. For interpretation purposes: larger magnitude ECs with bayesian-PP ≥ 0.95 are considered reliable. Test-retest reliability of ECs with larger magnitudes is higher. bayesian-PP is the conditional probability computed by PEB using Bayes rules after likelihood function and prior probability density of model parameters are taken into account. The higher the bayesian-PP, the greater confidence, although the concept of statistical significance does not directly apply. DCM experts recommend focusing on EC results with PP ≥ 0.90 or 0.95 as they reflect the most probable effects. In this report, we considered an EC finding reliable if bayesian-PP ≥ 0.95 (corresponding to a Bayes-factor of 20). For our PEB analyses in CON participants and our group comparison between AUD and CON participants, we focused our discussion on larger magnitude connectivities (|ec|≥0.3 hz, PP = 1).

*ec* effective connectivity, *pp* posterior probability, *l* left, and *r* right, *VMPFC* ventromedial prefrontal cortex, *vlpfc* ventrolateral prefrontal cortex, *amy* amygdala, *fg* fusform gyrus, *hth * hypothalamus

*Relationships between each EC and cumulative alcohol usage*: Linear regression analysis examining associations between EC and cumulative alcohol use was conducted in the total sample combining AUD and CON participants (Table 3). To highlight, some major EC results (beta ≥ 0.1 Hz, PP = 1) are shown in Fig. 3C. Specifically, the ECs from the VMPFC → the bilateral VLPFC, right VLPFC → bilateral FG, left FG → right VLPFC, right FG → HTN, and right AMY → left VLPFC were negatively associated with cumulative alcohol use and ECs from VMPFC → VMPFC, left VLPFC → right VLPFC and bilateral FG, left FG → right AMY and HTN, right AMY → VMPFC and left FG, and left AMY → HTN were positively associated with cumulative alcohol use (all PP’s = 1).

*Relationships between each EC and depression and anxiety scores*: Separate linear regression analyses examining EC associations with depression and anxiety scores were conducted in the combined sample of AUD and CON participants (Table 3). EC from the right VLPFC → the left FG (Fig. 3D)) was negatively associated with depression scores (beta=-0.0205, PP = 0.89). No associations between EC and anxiety scores were seen (no PP’s > 0.85).

*Supplemental and post-hoc analyses*: Results of our supplemental DCM analyses focused on sex/gender showed wide spread sex differences in multiple ECs and a number of ECs for which a group-by-sex interaction effect was observed (see supplemental Table S4-S5). Post-hoc linear regression analyses testing associations between EC and cumulative alcohol use, anxiety, and depression scores separately in AUD and CON groups are presented in supplemental Tables S6-S7.

## Discussion

Applying DCM to fMRI data obtained during an emotional face perception task in individuals with AUD and matched CON, we found evidence of aberrant EC response to emotional faces as a driving stimulus across both sides of a distributed face processing network in adults with AUD. Within the modeled neuronal network, our DCM analysis showed evidence of multi-network dysfunction with disrupted cortical-cortical, cortical-subcortical, and subcortical-subcortical ECs in both “top-down” and “bottom-up” pathways involving AMY, FG, and medial and lateral PFC regions among participants with AUD that differentiated them from CON participants and related to cumulative alcohol use and depression in the total sample. These network alterations are notable given that the AUD group did not differ from the CON group in brain activation or behavioral performance during the face processing task.

Our results in the CON group are generally consistent with previous DCM studies of healthy individuals during emotional face processing, some of which utilized slightly different sets of a priori nodes (Dima et al. 2011; Jamieson et al. 2021; Ma et al. 2020). Notably, each of these studies showed modulation in ECs between VLPFC, FG, and AMY regions in common, suggesting that these ECs are largely robust across specific localizations of nodes, analytic design choices, and patient populations. In the CON group, the ECs from VMPFC to bilateral VLPFC, AMY, and FG were all positive, suggesting a crucial role of the VMPFC in the CON group, consistent with the finding that VMPFC is important for normative responding to aversive stimuli and emotion regulation (Buhle et al. 2014).

**Fig. 4:**
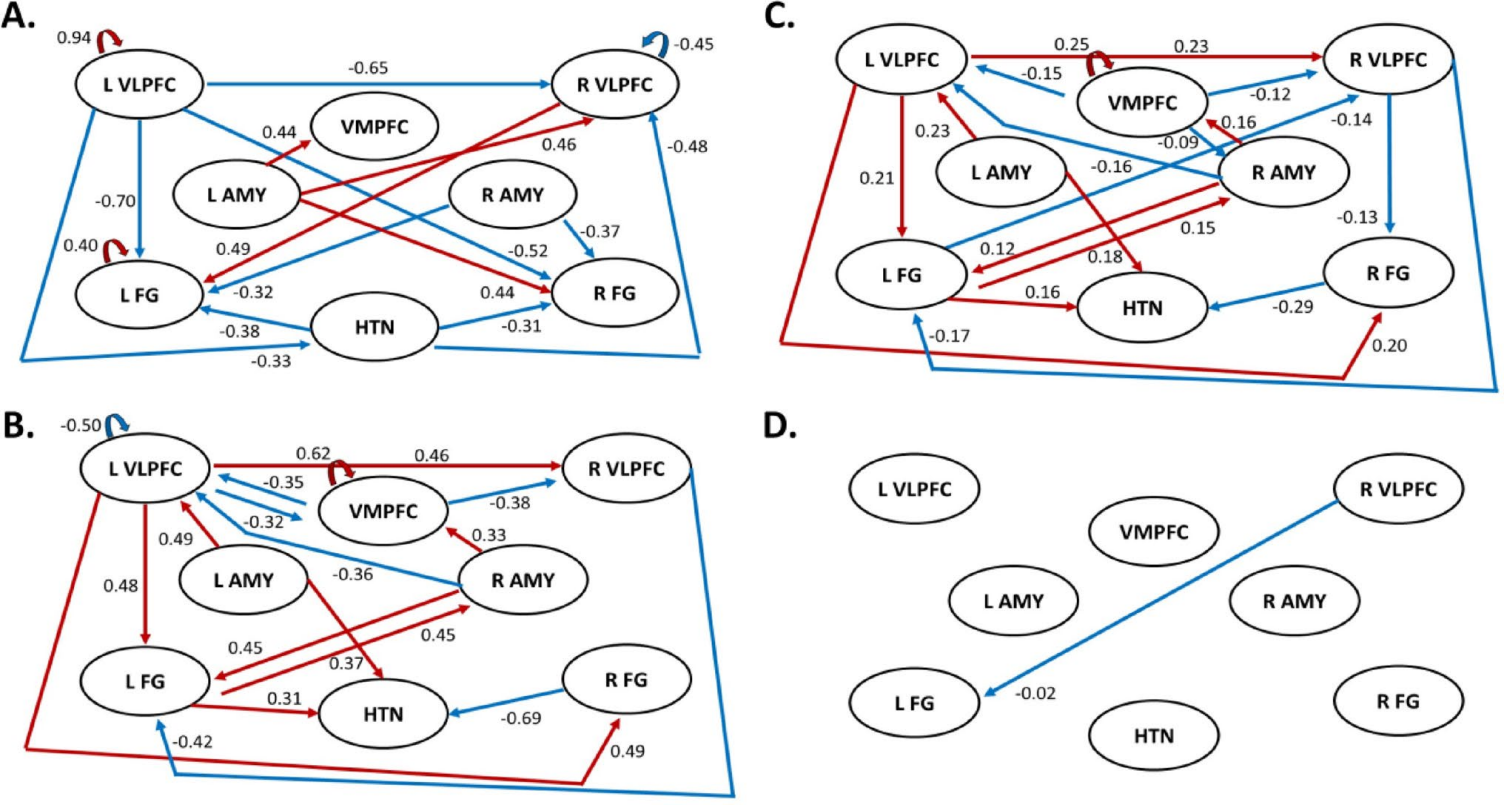
Schematic Circuit Diagram of Emotional Face Processing Network Showing DCM Analysis Findings. **A**: Circuit Diagram Showing DCM Analysis Results of EC for emotional faces minus shapes contrast in CON participants (*n* = 70, beta: *≥*0.3 Hz, posterior probability [PP] > 0.95). This diagram represents EC during emotional face processing in healthy adults. **B**: Circuit Diagram Showing DCM Analysis Results of EC Group Differences for AUD minus CON participants (*N* = 140, *n* = 70 per group, beta: *≥*0.3 Hz, PP > 0.95) for emotional face minus shape contrast. This diagram represents EC modulation as a function of AUD diagnostic status reflecting how EC differs between AUD and CON groups. **C**: Circuit diagram showing DCM analysis results of linear regression between EC outcomes (emotional faces minus shapes) and cumulative alcohol use in the full sample collapsed across AUD and CON participants (*N* = 140, beta: *≥*0.1 Hz, PP > 0.95). **D**: Circuit diagram showing DCM analysis results of linear regression between EC outcomes (emotional faces minus shapes) and AASR depression scores in the full sample collapsed across AUD and CON participants (*N* = 140, PP = 0.89). In the figure, red lines represent EC between nodes that is greater in response to emotional faces compared to shapes in A, greater in AUD compared to CON participants in B, and positively association with cumulative alcohol use in C. Blue lines represent EC between nodes that is lower (more negative) in response to emotional faces compared to shapes in A, lower in AUD compared to CON participants in B, and negatively associated with cumulative alcohol use and depression scores in C and D, respectively. Strength of EC in Hz is presented next to each line. *AUD* alcohol use disorder; *CON* control; *VMPFC* ventromedial prefrontal cortex; *VLPFC* ventrolateral prefrontal cortex; *AMY* amygdala; *FG* Fusiform Gyrus; *HTN* Hypothalamus; *L* Left; *R* Right

In our between-group analysis, we found modulatory EC differences between AUD and CON groups across multiple regions. (see Figs. 3B and 4B). Relative to the CON group, the AUD group had lower ECs from the lower EC from VMPFC → bilateral VLPFC, left VLPFC → left VLPFC and VMPFC, right VLPFC → left FG, right FG → HTN, and right AMY → left VLPFC and these ECs were negatively associated with the cumulative alcohol use. These findings are in parallel with previous studies showing that the function of the VMPFC is disrupted in AUD (Seo et al. 2013; Silvers et al. 2017). On the other hand, the AUD group showed greater ECs within VMPFC and from left VLPFC → right VLPFC and bilateral FG, left FG → right AMY and HTN, right AMY → VMPFC and left FG, and left AMY → HTN than the CON group and these ECs were positively associated with the cumulative alcohol use. VMPFC and VLPFC (especially left VLPFC) are key regions in regulating emotion (Berboth and Morawetz 2021; Nelson and Guyer 2011; Suzuki et al. 2020). Reduced VMPFC regulation facilitates the VLPFC-related regulation (Silvers et al. 2017). Thus, these enhanced ECs from the left VLPFC suggest that the AUD participants’ brains may be less efficient and require additional top-down resource allocation (left VLPFC input) to achieve similar behavioral performance to CON on the task, perhaps by minimizing potential interference by more salient (facial) images. The FG plays a specialized role in facial perception and sensitivity (Grill-Spector et al. 2004; Puce et al. 1995), especially for fearful faces (Wang et al. 2017; Wudarczyk et al. 2016). Thus, the greater left VLPFC → bilateral FG ECs may reflect the increased control of the left VLPFC over the sensitivity to negative facial expression in the AUD group. Previous studies have shown reduced FG activation during emotion regulation (Silvers et al. 2017) and PFC-FG connectivity in AUD (Oberlin et al. 2012).

Our AUD-CON group comparison results align with previous EC studies that show multi-network dysfunction in AUD (Arias et al. 2021; Radoman et al. 2024; Song et al. 2021; Stout et al. 2023), although this literature is notably sparse. Focusing on our within-network results, we found EC differences within the left VLPFC and the VMPFC that were lower and greater, respectively, in the AUD vs. CON group. Our between-network results showed a complex pattern of EC differences between VLPFC, VMPFC, and AMY. These brain regions represent key nodes of the DMN, SN, FPN, and negative affective (NA) network (Williams 2016). At the network-level, our results align with previous FC studies that show DMN hyperconnectivity, FPN hypoconnectivity, and altered FC between DMN, FPN, and SN in AUD patients (Beck et al. 2012; Camchong et al. 2013; Canessa et al. 2021; Chanraud et al. 2011; Courtney et al. 2013; Muller-Oehring et al. 2013, 2015) with some of these alterations associated with relapse risk following AUD treatment.

Taken together, our results are consistent with prior research showing multi-network dysfunction during emotional face processing in AUD. Our work extends this line of research, showing for the first time the directionality of these circuit alterations, and revealing that directional connectivity in both “bottom-up” emotion generation and perception and “top-down” emotion regulation circuits is aberrant in AUD. It is important to note that group-level EC differences emerged in the context of normal behavioral responding and activation during this task. EC results observed may reflect both pathophysiological processes and compensatory responses in AUD. Additional research is warranted to further investigate putative pathophysiological and compensatory process related to emotion processing in AUD using sensitive measures of emotion recognition/awareness and regulation and multivariate analyses to determine if aberrant ECs in AUD relate to real-world social/emotional skills deficits.

Partially consistent with our hypotheses, results from the DCM analysis showed some evidence for modulatory changes in EC within and between circuits involving VLPFC, VMPFC, and AMY regions that differed between AUD and CON groups, but generally pointed to a more complex presentation of directional relationships and connectivity differences than originally hypothesized. One example of this is as follows: Results from direct connections between pairs of brain regions did not support our hypothesis that the AUD group would have lower EC from VMPFC and VLPFC → AMY than the CON group, as a manifestation of poorer top-down frontocortical control. However, it is necessary to note that in the context of EC, the causal influence which one node exerts over another can also be indirect, passing through a third node (Colombo and Weinberger 2018). Indeed, our EC results provided some evidence for an indirect pathway for VLPFC to modulate change in AMY through FG via a three-node circuit that showed lower EC from right VLPFC → left FG (-0.42 Hz) and greater EC from left FG → right AMY (0.45 Hz) in the AUD compared to CON group. These findings should be considered in tandem with our results showing lower within-network EC in the left VLPFC for the AUD group. Thus, despite not showing directional modulation of VLPFC on AMY, when taken together, these results are consistent with individuals with AUD having impairments in top-down control over the AMY during emotional face processing, albeit through indirect pathways. Future research will require more complex systems/circuit level analyses to elucidate these complex relationships between multiple nodes and systems for disorders with multi-network dysfunction like AUD.

Consistent with our hypothesis, EC differences from the left AMY → right HTN were greater in the AUD compared to the CON group. AMY, HTN, and midbrain structures (e.g., periaqueductal gray and medulla oblongata) are three interconnected brain regions forming a circuit that is involved in fear responding, autonomic changes, and hypothalamic-pituitary-adrenal (HPA)-axis brain stress system reactivity (Steimer 2002). In particular, connectivity between these regions are implicated in autonomic activation and rapid arousal during periods of perceived threat (Liddell et al. 2005); and conversely can be down-regulated by the VMPFC, which plays a key role in controlling this circuit (Thayer et al. 2012), and may be impaired in AUD. Thus, the greater EC modulatory change in the left AMY → HTN observed in the AUD group suggests hyperconnectivity within the extended amygdala/HPA-axis stress response system and enhanced arousal and autonomic response in AUD during emotional face processing (Seo et al. 2024).

We also found some evidence for right-to-left shift in lateralization for ECs in AUD vs. CON groups in three circuits involving VLPFC, FG, AMY, and HTN. Lateralization is not surprising, given the asymmetric functional specialization of cerebral hemispheres conserved across species, although right-vs-left hemispheric differences could signify asymmetric connectivities in AUD that are meaningful for social/emotional behavior. For the VLPFCL → FG circuit: Participants in the AUD group showed lower EC from the right VLPFC → left FG and higher EC from the left VLPFC → left FG compared to those in the CON group. This is the reverse of the pattern of ECs observed in the CON group. These results suggest that left lateral PFC modulatory control over left FG may represent a compensatory mechanism for overcoming deficits in emotion face processing in AUD individuals. The interpretation that the left VLPFC → left FG increase in EC is compensatory for the right VLPFC → left FG decrease in EC is consistent with previous studies showing the compensatory role of left VLPFC for right VLPFC function (see Tan et al. (2007) and Holler-Wallscheid et al. (2017) for reviews on bilateral PFC compensatory mechanism hypothesis). Laterality findings in the other two circuits mirrored each other. ECs in the CON group from AMY → left VLPFC and from FG → HTN were not observed. AUD-CON group comparison analyses showed a right-to-left shift in laterality of ECs in both circuits as a function of AUD diagnostic status, whereby the AUD group showed greater modulatory changes in the left AMY → left VLPFC and left FG → right HTN and lesser modulatory changes in the right AMY → left VLPFC and right FG → right HTN, respectively. Our lateralization findings are consistent with the behavioral inhibition/activation (BIS/BAS) model of motivated behavior which posits left-hemispheric dominant approach motivational tendencies among individuals with SUDs (Demaree et al. 2005). Hemispheric lateralization of emotion processing and motivational networks have long been described in the literature (see Palomero-Gallagher and Amunts (2022), with recent work suggesting AMY and FG lateralization of certain sub-functions related to feature processing and language (Berboth and Morawetz 2021; Meng et al. 2012; Sergerie et al. 2008) that may be disrupted in AUD.

Our linear regression analyses showed convergent EC results in a right VLPFC → left FG circuit that was related to both depression and alcohol use in the sample. Specifically, we found that EC from the right VLPFC → left FG was negatively associated with depression severity (PP = 0.89) and cumulative alcohol use (PP = 1) in the total sample, and also showed AUD-CON group differences (AUD-CON=-0.42 Hz, PP = 1). While the PP of 0.89 for depression falls below our threshold of PP > 0.95, it is very close to PP = 0.9 (a level considered meaningful, as recommended by the DCM authors detailed in our Methods section). Further investigation into the temporal coupling between alcohol use and depressive symptoms and how variations in alcohol-depression phenotypes differ with regard to EC are needed. Impaired VLPFC modulation over FG during emotional face processing among people with greater alcohol use and depression severity could reflect impairments in PFC-drive emotion regulation or deficits in emotion recognition related to impaired SIP and attentional allocation towards negative emotional face stimuli. Both of these explanatory models point to impaired top-down lateral PFC control processes in this population and suggest that these EC alterations may be dually implicated in AUD and depression risk and might reflect a transdiagnostic sociobiological marker shared by these conditions (Voon et al. 2020). This result is consistent with findings from previous fMRI studies showing dysfunction in FG (Ho et al. 2016) and alteration in FC and EC between the FG and lateral PFC regions in mood disorders (Sheng et al. 2024; Willinger et al. 2022). Additional research is warranted to determine if R-VLPFCL-FG represents a putative neural treatment target for individuals with co-occurring/comorbid depression and AUD.

This study has several limitations. As our analyses were conducted using publicly available HCP data, we were reliant on HCP methodology, sampling, measurement, task selection, and fMRI scanning parameters and preprocessing techniques. Thus, our study reflects HCPs strengths and weaknesses. For example, the face-matching task required minimal cognitive load, and may not have driven relevant regulatory systems. Of interest for future studies would be a more complex task that required greater demands to avoid attentional capture by salient/emotional faces (Bjork et al. 2021). AUD diagnoses and most substance-related and psychological variables used in this study were assessed via self-report with the SSAGA and ASR. These forms of data lend themselves to recall or social desirability bias. Precision and representativeness of AUD diagnoses is also a limitation of our study. Participants with AUD were neither clinically referred nor recruited based on their alcohol use; although, the HCP did allow for inclusion of persons who met SUD criteria, provided those individuals had never been in SUD treatment. Though current vs. lifetime AUD history data was not provided in the publicly available dataset, nearly all AUD participants self-reported hazardous drinking within the past 12 months, suggesting some degree of recent AUD symptomatology. As the focus of our study was on EC differences related to AUD and alcohol, we chose to exclude individuals who met criteria for a non-alcohol SUD or who reported more than experimental use of other drugs. While this enabled us to examine unique EC associations with AUD, it may have also impacted the generalizability of the sample. Investigations into EC differences related to alcohol and other drug co-use is warranted in subgroups who engage in poly-drug use or who have comorbid AUD and other SUD. This line of research may help to clarify whether the EC differences in AUD identified here are AUD-specific or reflect general EC alterations seen across SUDs regardless of type (Klugah-Brown et al. 2020). Specifically related to cannabis use: We used a positive THC urine test as an exclusion criterion to minimize potential confounding effects of recent or chronic cannabis use on brain activation and connectivity. Consistent with this exclusion criterion, a recent study using the HCP dataset (Gowin et al. 2025) found that recent cannabis use, as indicated by a positive THC urine test, was associated with poorer task-related performance and alterations in brain function. While this exclusion criterion helps control for confounding effects, it may have also excluded individuals who used cannabis infrequently and had residual cannabinoids in their urine from a recent use episode, reflective of the non-specificity of urine cannabis testing in this regard. This limitation arises from the dataset being not specifically designed for cannabis research and lacking detailed measures of recent cannabis use. Despite this limitation, we maintained sufficiently large sample sizes (*n* = 70 per group) after exclusion, ensuring adequate statistical power for our analyses.

Our study focused on DCM applied to fMRI data. While DCM is a well-established approach for modeling EC, we acknowledge that its predefined model may limit the detection of unexpected or emergent pathways. Structural connectivity data can provide valuable insights for validating predefined DCM models; however, conducting a structural connectivity analysis is beyond the scope of the current study, despite the availability of diffusion MRI data for most included participants. Integrating DCM-based EC analysis with diffusion MRI-derived structural connectivity is an important direction for our future research.

Regarding the fMRI analyses, the modeled neuronal circuit/network is relatively small, but this was necessitated by computational constraints in DCM discussed previously in Ma et al. (2018). The DCM nodes used in this study were a priori selected from previous fMRI and FC studies of face processing and AUD and further constrained by the actual sample-specific brain activations. This DCM node selection approach that uses task-evoked activation fMRI data from the AUD and CON groups, could hypothetically result in overfitting, although the alternative approach of using data from held out HCP subjects to guide DCM node creation also carries limitations, as it may not reflect activation patterns representative of the included groups, leading to biased or unbalanced DCM node selection and underfitting (Seghier et al. 2010). Additionally, given DCM node selection issues, it is possible that other altered network connectivities were not identified because the connecting regions were not included as DCM nodes. Another limitation of this study could be related to head motion which can impact FC outcomes (Ikari et al. 2012); although this is unlikely for the current report, as HCP took several steps during data collection and processing to mitigate the effects of head motion (Marcus et al. 2013), and our analysis showed no significant group differences between AUD and CON on FD. Lastly, the cross-sectional design of this study precluded our ability to discern which EC alterations represent predispositional EC differences conveying risk for AUD and which represent neuroplastic/neuroadaptive EC changes that develop as a consequence of cumulative heavy drinking episodes (Baranger et al. 2020). Longitudinal studies incorporating neuroimaging and phenotypic measures, such as the Adolescent Brain Cognitive Development (ABCD) study, are needed to further disentangle potential etiological causes of the EC differences observed here (Cosgrove 2016; Karcher and Barch 2021).

## Conclusions

We observed EC differences in cortical-cortical and cortical-subcortical circuits during emotional face processing that varied as a function of AUD diagnostic status and related to cumulative alcohol exposure and depression. Results from this study corroborate and extend findings from previous fMRI/FC studies in AUD by showing putative directional relationships. EC differences from our study were observed across both sides of a distributed face processing network that included medial and lateral PFC regions, AMY, HTN, and FG. Our EC analyses showed a complex pattern of directional relationships that encompassed top-down and bottom-up, within and between network, and laterally distinct EC differences. Collectively, these results suggest multi-network dysfunction during emotional face processing in AUD. While not completely aligned with classic models of AUD pathophysiology, our findings lend further support for a neurobiological model of AUD that includes impaired “top-down” emotion control and aberrant “bottom-up” emotion perception signaling during face processing that could explain some of the variance related to deficits of emotion perception, awareness, and regulation in this population. Findings from this study provide a strong initial framework on AUD-related circuit alterations in emotion perception and regulation brain networks to inform next stage EC studies and circuit-targeted intervention development studies for patients with AUD and mood disorders.

## Electronic supplementary material

Below is the link to the electronic supplementary material.

Supplementary Material 1

## Data Availability

No datasets were generated or analysed during the current study.

## References

[CR1] Achenbach TM (2009) The Achenbach system of empirically based assessment (ASEBA): development, findings, theory, and applications. University of Vermont, Research Center of Children, Youth & Families, Burlington

[CR2] Arias AJ, Ma L, Bjork JM, Hammond CJ, Zhou Y, Snyder A, Moeller FG (2021) Altered effective connectivity of the reward network during an incentive-processing task in adults with alcohol use disorder. Alcohol Clin Exp Res 45:1563–1577 34120362 10.1111/acer.14650 PMC8742221

[CR3] Arnsten AF (2009) Stress signalling pathways that impair prefrontal cortex structure and function. Nat Rev Neurosci 10:410–422 19455173 10.1038/nrn2648 PMC2907136

[CR4] Babor TF, Caetano R (2006) Subtypes of substance dependence and abuse: implications for diagnostic classification and empirical research. Addiction 101(Suppl 1):104–110 16930166 10.1111/j.1360-0443.2006.01595.x

[CR5] Baranger DAA, Demers CH, Elsayed NM, Knodt AR, Radtke SR, Desmarais A, Few LR, Agrawal A, Heath AC, Barch DM, Squeglia LM, Williamson DE, Hariri AR, Bogdan R (2020) Convergent evidence for predispositional effects of brain Gray matter volume on alcohol consumption. Biol Psychiatry 87:645–655 31699293 10.1016/j.biopsych.2019.08.029 PMC7412715

[CR6] Barch DM, Burgess GC, Harms MP, Petersen SE, Schlaggar BL, Corbetta M, Glasser MF, Curtiss S, Dixit S, Feldt C, Nolan D, Bryant E, Hartley T, Footer O, Bjork JM, Poldrack R, Smith S, Johansen-Berg H, Snyder AZ, Van Essen DC, Consortium WU-MH (2013) Function in the human connectome: task-fMRI and individual differences in behavior. NeuroImage 80:169–189 23684877 10.1016/j.neuroimage.2013.05.033 PMC4011498

[CR7] Baroncini M, Jissendi P, Balland E, Besson P, Pruvo JP, Francke JP, Dewailly D, Blond S, Prevot V (2012) MRI atlas of the human hypothalamus. NeuroImage 59:168–180 21777680 10.1016/j.neuroimage.2011.07.013

[CR8] Beck A, Wustenberg T, Genauck A, Wrase J, Schlagenhauf F, Smolka MN, Mann K, Heinz A (2012) Effect of brain structure, brain function, and brain connectivity on relapse in alcohol-dependent patients. Arch Gen Psychiatry 69:842–852 22868938 10.1001/archgenpsychiatry.2011.2026

[CR9] Berboth S, Morawetz C (2021) Amygdala-prefrontal connectivity during emotion regulation: A meta-analysis of Psychophysiological interactions. Neuropsychologia 153:107767 33516732 10.1016/j.neuropsychologia.2021.107767

[CR10] Bjork JM, Keyser-Marcus L, Vassileva J, Ramey T, Houghton DC, Moeller FG (2021) Social information processing in substance use disorders: insights from an emotional go-nogo task. Front Psychiatry 12:672488 34122188 10.3389/fpsyt.2021.672488 PMC8193089

[CR11] Bucholz KK, Cadoret R, Cloninger CR, Dinwiddie SH, Hesselbrock VM, Nurnberger JI Jr., Reich T, Schmidt I, Schuckit MA (1994) A new, semi-structured psychiatric interview for use in genetic linkage studies: a report on the reliability of the SSAGA. J Stud Alcohol 55:149–158 8189735 10.15288/jsa.1994.55.149

[CR12] Buhle JT, Silvers JA, Wager TD, Lopez R, Onyemekwu C, Kober H, Weber J, Ochsner KN (2014) Cognitive reappraisal of emotion: a meta-analysis of human neuroimaging studies. Cereb Cortex 24:2981–2990 23765157 10.1093/cercor/bht154 PMC4193464

[CR13] Camchong J, Stenger A, Fein G (2013) Resting-state synchrony during early alcohol abstinence can predict subsequent relapse. Cereb Cortex 23:2086–2099 22819968 10.1093/cercor/bhs190 PMC3729195

[CR14] Canessa N, Basso G, Carne I, Poggi P, Gianelli C (2021) Increased decision latency in alcohol use disorder reflects altered resting-state synchrony in the anterior salience network. Sci Rep 11:19581 34599268 10.1038/s41598-021-99211-1 PMC8486863

[CR15] Carretie L, Albert J, Lopez-Martin S, Tapia M (2009) Negative brain: an integrative review on the neural processes activated by unpleasant stimuli. Int J Psychophysiol 71:57–63 18727941 10.1016/j.ijpsycho.2008.07.006

[CR16] Chanraud S, Pitel AL, Pfefferbaum A, Sullivan EV (2011) Disruption of functional connectivity of the default-mode network in alcoholism. Cereb Cortex 21:2272–2281 21368086 10.1093/cercor/bhq297 PMC3169657

[CR17] Clark US, Oscar-Berman M, Shagrin B, Pencina M (2007) Alcoholism and judgments of affective stimuli. Neuropsychology 21:346–362 17484598 10.1037/0894-4105.21.3.346 PMC4087115

[CR18] Colombo M, Weinberger N (2018) Discovering brain mechanisms using network analysis and causal modeling. Minds Mach (Dordr) 28:265–286 30996522 10.1007/s11023-017-9447-0 PMC6438494

[CR19] Cosgrove KP (2016) A need for longitudinal studies in the addiction field. Biol Psychiatry 80:174–175 27402471 10.1016/j.biopsych.2016.05.018

[CR20] Courtney KE, Ghahremani DG, Ray LA (2013) Fronto-striatal functional connectivity during response Inhibition in alcohol dependence. Addict Biol 18:593–604 23240858 10.1111/adb.12013 PMC3683582

[CR21] Demaree HA, Everhart DE, Youngstrom EA, Harrison DW (2005) Brain lateralization of emotional processing: historical roots and a future incorporating dominance. Behav Cogn Neurosci Rev 4:3–20 15886400 10.1177/1534582305276837

[CR22] Dima D, Stephan KE, Roiser JP, Friston KJ, Frangou S (2011) Effective connectivity during processing of facial affect: evidence for multiple parallel pathways. J Neurosci 31:14378–14385 21976523 10.1523/JNEUROSCI.2400-11.2011 PMC6623650

[CR23] Drysdale AT, Grosenick L, Downar J, Dunlop K, Mansouri F, Meng Y, Fetcho RN, Zebley B, Oathes DJ, Etkin A, Schatzberg AF, Sudheimer K, Keller J, Mayberg HS, Gunning FM, Alexopoulos GS, Fox MD, Pascual-Leone A, Voss HU, Casey BJ, Dubin MJ, Liston C (2017) Erratum: Resting-state connectivity biomarkers define neurophysiological subtypes of depression. Nat Med 23:264 28170383 10.1038/nm0217-264d PMC5639473

[CR24] Etkin A, Wager TD (2007) Functional neuroimaging of anxiety: a meta-analysis of emotional processing in PTSD, social anxiety disorder, and specific phobia. Am J Psychiatry 164:1476–1488 17898336 10.1176/appi.ajp.2007.07030504 PMC3318959

[CR25] Foisy ML, Philippot P, Verbanck P, Pelc I, van der Straten G, Kornreich C (2005) Emotional facial expression decoding impairment in persons dependent on multiple substances: impact of a history of alcohol dependence. J Stud Alcohol 66:673–681 16329459 10.15288/jsa.2005.66.673

[CR26] Frassle S, Stephan KE (2022) Test-retest reliability of regression dynamic causal modeling. Netw Neurosci 6:135–160 35356192 10.1162/netn_a_00215 PMC8959103

[CR27] Freeman CR, Wiers CE, Sloan ME, Zehra A, Ramirez V, Wang GJ, Volkow ND (2018) Emotion recognition biases in alcohol use disorder. Alcohol Clin Exp Res 10.1111/acer.13802 29975424

[CR30] Friston KJ, Penny W (2003) Posterior probability maps and SPMs. NeuroImage 19:1240–1249 12880849 10.1016/s1053-8119(03)00144-7

[CR28] Friston KJ, Harrison L, Penny W (2003) Dynamic causal modelling. Neuroimage 19, 1273–1302. PMID: 12948688 10.1016/s1053-8119(03)00202-7 12948688

[CR29] Friston KJ, Litvak V, Oswal A, Razi A, Stephan KE, van Wijk BC, Ziegler G, Zeidman P (2016) Bayesian model reduction and empirical Bayes for group (DCM) studies. NeuroImage 128:413–431 26569570 10.1016/j.neuroimage.2015.11.015 PMC4767224

[CR31] Friston KJ, Preller KH, Mathys C, Cagnan H, Heinzle J, Razi A, Zeidman P (2017) Dynamic causal modelling revisited. Neuroimage. 10.1016/j.neuroimage.2017.02.045 PMC6693530 28219774

[CR32] Friston KJ, Preller KH, Mathys C, Cagnan H, Heinzle J, Razi A, Zeidman P (2019) Dynamic causal modelling revisited. NeuroImage 199:730–744 28219774 10.1016/j.neuroimage.2017.02.045 PMC6693530

[CR33] Glasser MF, Sotiropoulos SN, Wilson JA, Coalson TS, Fischl B, Andersson JL, Xu J, Jbabdi S, Webster M, Polimeni JR, Van Essen DC, Jenkinson M, Consortium WU-MH (2013) The minimal preprocessing pipelines for the human connectome project. NeuroImage 80:105–124 23668970 10.1016/j.neuroimage.2013.04.127 PMC3720813

[CR34] Gorka SM, Fitzgerald DA, King AC, Phan KL (2013) Alcohol attenuates amygdala-frontal connectivity during processing social signals in heavy social drinkers: a preliminary pharmaco-fMRI study. Psychopharmacology 229:141–154 23584670 10.1007/s00213-013-3090-0 PMC3740023

[CR35] Gowin JL, Ellingson JM, Karoly HC, Manza P, Ross JM, Sloan ME, Tanabe JL, Volkow ND (2025) Brain function outcomes of recent and lifetime cannabis use. JAMA Netw Open 8:e2457069 39874032 10.1001/jamanetworkopen.2024.57069 PMC11775743

[CR36] Grill-Spector K, Knouf N, Kanwisher N (2004) The fusiform face area subserves face perception, not generic within-category identification. Nat Neurosci 7:555–562 15077112 10.1038/nn1224

[CR37] Hariri AR, Tessitore A, Mattay VS, Fera F, Weinberger DR (2002) The amygdala response to emotional stimuli: a comparison of faces and scenes. NeuroImage 17:317–323 12482086 10.1006/nimg.2002.1179

[CR38] Haxby JV, Hoffman EA, Gobbini MI (2000) The distributed human neural system for face perception. Trends Cogn Sci 4:223–233 10827445 10.1016/s1364-6613(00)01482-0

[CR39] Heinz A, Wrase J, Kahnt T, Beck A, Bromand Z, Grusser SM, Kienast T, Smolka MN, Flor H, Mann K (2007) Brain activation elicited by affectively positive stimuli is associated with a lower risk of relapse in detoxified alcoholic subjects. Alcohol Clin Exp Res 31:1138–1147 17488322 10.1111/j.1530-0277.2007.00406.x

[CR40] Hillebrandt H, Friston KJ, Blakemore SJ (2014) Effective connectivity during animacy perception–dynamic causal modelling of human connectome project data. Sci Rep 4:6240 25174814 10.1038/srep06240 PMC4150124

[CR41] Ho TC, Zhang S, Sacchet MD, Weng H, Connolly CG, Henje Blom E, Han LK, Mobayed NO, Yang TT (2016) Fusiform gyrus dysfunction is associated with perceptual processing efficiency to emotional faces in adolescent depression: A model-based approach. Front Psychol 7:40 26869950 10.3389/fpsyg.2016.00040 PMC4740953

[CR42] Holler-Wallscheid MS, Thier P, Pomper JK, Lindner A (2017) Bilateral recruitment of prefrontal cortex in working memory is associated with task demand but not with age. Proc Natl Acad Sci U S A 114:E830–E839 28096364 10.1073/pnas.1601983114 PMC5293052

[CR43] Ikari Y, Nishio T, Makishi Y, Miya Y, Ito K, Koeppe RA, Senda M (2012) Head motion evaluation and correction for PET scans with 18F-FDG in the Japanese alzheimer’s disease neuroimaging initiative (J-ADNI) multi-center study. Ann Nucl Med 26:535–544 22763629 10.1007/s12149-012-0605-4

[CR44] Imtiaz S, Loheswaran G, Le Foll B, Rehm J (2018) Longitudinal alcohol consumption patterns and health-related quality of life: results from the National epidemiologic survey on alcohol and related conditions. Drug Alcohol Rev 37:48–55 28294429 10.1111/dar.12503

[CR45] Jamieson AJ, Davey CG, Harrison BJ (2021) Differential modulation of effective connectivity in the brain’s extended face processing system by fearful and sad facial expressions. eNeuro 8 10.1523/ENEURO.0380-20.2021 PMC8174049 33658311

[CR46] Joormann J, Stanton CH (2016) Examining emotion regulation in depression: A review and future directions. Behav Res Ther 86:35–49 27492851 10.1016/j.brat.2016.07.007

[CR47] Karcher NR, Barch DM (2021) The ABCD study: Understanding the development of risk for mental and physical health outcomes. Neuropsychopharmacology 46:131–142 32541809 10.1038/s41386-020-0736-6 PMC7304245

[CR48] Kenwood MM, Kalin NH, Barbas H (2022) The prefrontal cortex, pathological anxiety, and anxiety disorders. Neuropsychopharmacology 47:260–275 34400783 10.1038/s41386-021-01109-z PMC8617307

[CR49] Klugah-Brown B, Di X, Zweerings J, Mathiak K, Becker B, Biswal B (2020) Common and separable neural alterations in substance use disorders: A coordinate-based meta-analyses of functional neuroimaging studies in humans. Hum Brain Mapp 41:4459–4477 32964613 10.1002/hbm.25085 PMC7555084

[CR50] Kong L, Chen K, Tang Y, Wu F, Driesen N, Womer F, Fan G, Ren L, Jiang W, Cao Y, Blumberg HP, Xu K, Wang F (2013) Functional connectivity between the amygdala and prefrontal cortex in medication-naive individuals with major depressive disorder. J Psychiatry Neurosci 38:417–422 24148846 10.1503/jpn.120117 PMC3819156

[CR51] Koob GF (2017) The dark side of addiction: the Horsley Gantt to Joseph Brady connection. J Nerv Ment Dis 205:270–272 27356121 10.1097/NMD.0000000000000551 PMC6135258

[CR52] Koob GF, Volkow ND (2016) Neurobiology of addiction: a neurocircuitry analysis. Lancet Psychiatry 3:760–773 27475769 10.1016/S2215-0366(16)00104-8 PMC6135092

[CR53] Kornreich C, Blairy S, Philippot P, Hess U, Noel X, Streel E, Le Bon O, Dan B, Pelc I, Verbanck P (2001) Deficits in recognition of emotional facial expression are still present in alcoholics after mid- to long-term abstinence. J Stud Alcohol 62:533–542 11513232 10.15288/jsa.2001.62.533

[CR54] Krause FC, Linardatos E, Fresco DM, Moore MT (2021) Facial emotion recognition in major depressive disorder: A meta-analytic review. J Affect Disord 293:320–328 34229285 10.1016/j.jad.2021.06.053 PMC8457509

[CR55] Kwako LE, Momenan R, Litten RZ, Koob GF, Goldman D (2016) Addictions neuroclinical assessment: A neuroscience-based framework for addictive disorders. Biol Psychiatry 80:179–189 26772405 10.1016/j.biopsych.2015.10.024 PMC4870153

[CR56] Le Berre AP (2019) Emotional processing and social cognition in alcohol use disorder. Neuropsychology 33:808–821 31448948 10.1037/neu0000572 PMC6711390

[CR57] Lemaire JJ, Nezzar H, Sakka L, Boirie Y, Fontaine D, Coste A, Coll G, Sontheimer A, Sarret C, Gabrillargues J, De Salles A (2013) Maps of the adult human hypothalamus. Surg Neurol Int 4, S156–163 10.4103/2152-7806.110667 PMC3654779 23682342

[CR58] Liddell BJ, Brown KJ, Kemp AH, Barton MJ, Das P, Peduto A, Gordon E, Williams LM (2005) A direct brainstem-amygdala-cortical ‘alarm’ system for subliminal signals of fear. NeuroImage 24:235–243 15588615 10.1016/j.neuroimage.2004.08.016

[CR62] Ma L, Steinberg JL, Hasan KM, Narayana PA, Kramer LA, Moeller FG (2012) Working memory load modulation of parieto-frontal connections: evidence from dynamic causal modeling. Hum Brain Mapp 33:1850–1867 21692148 10.1002/hbm.21329 PMC3179779

[CR63] Ma L, Steinberg JL, Hasan KM, Narayana PA, Kramer LA, Moeller FG (2014) Stochastic dynamic causal modeling of working memory connections in cocaine dependence. Hum Brain Mapp 35:760–778 23151990 10.1002/hbm.22212 PMC4440319

[CR60] Ma L, Steinberg JL, Bjork JM, Keyser-Marcus L, Vassileva J, Zhu M, Ganapathy V, Wang Q, Boone EL, Ferre S, Bickel WK, Gerard Moeller F (2018) Fronto-striatal effective connectivity of working memory in adults with cannabis use disorder. Psychiatry Res Neuroimaging 278:21–34 29957349 10.1016/j.pscychresns.2018.05.010 PMC6953485

[CR61] Ma L, Steinberg JL, Bjork JM, Wang Q, Hettema JM, Abbate A, Moeller FG (2020) Altered effective connectivity of central autonomic network in response to negative facial expression in adults with cannabis use disorder. Biol Psychiatry Cogn Neurosci Neuroimaging 5:84–96 31345781 10.1016/j.bpsc.2019.05.013 PMC8598077

[CR59] Ma L, Hettema JM, Cousijn J, Bjork JM, Steinberg JL, Keyser-Marcus L, Woisard K, Lu Q, Roberson-Nay R, Abbate A, Moeller FG (2021) Resting-state directional connectivity and anxiety and depression symptoms in adult cannabis users. Biol Psychiatry Cogn Neurosci Neuroimaging 6:545–555 33388293 10.1016/j.bpsc.2020.09.015 PMC8968690

[CR64] Marcus D, Harwell J, Olsen T, Hodge M, Glasser M, Prior F, Jenkinson M, Laumann T, Curtiss S, Van Essen D (2011) Informatics and Data Mining Tools and Strategies for the Human Connectome Project. Frontiers in Neuroinformatics 5 10.3389/fninf.2011.00004 PMC3127103 21743807

[CR65] Marcus DS, Harms MP, Snyder AZ, Jenkinson M, Wilson JA, Glasser MF, Barch DM, Archie KA, Burgess GC, Ramaratnam M, Hodge M, Horton W, Herrick R, Olsen T, McKay M, House M, Hileman M, Reid E, Harwell J, Coalson T, Schindler J, Elam JS, Curtiss SW, Van Essen DC, Consortium WU-MH (2013) Human connectome project informatics: quality control, database services, and data visualization. NeuroImage 80:202–219 23707591 10.1016/j.neuroimage.2013.05.077 PMC3845379

[CR66] Marinkovic K, Oscar-Berman M, Urban T, O’Reilly CE, Howard JA, Sawyer K, Harris GJ (2009) Alcoholism and dampened Temporal limbic activation to emotional faces. Alcohol Clin Exp Res 33:1880–1892 19673745 10.1111/j.1530-0277.2009.01026.x PMC3543694

[CR67] Meng M, Cherian T, Singal G, Sinha P (2012) Lateralization of face processing in the human brain. Proc Biol Sci 279:2052–2061 22217726 10.1098/rspb.2011.1784 PMC3311882

[CR69] Muller-Oehring EM, Jung YC, Sullivan EV, Hawkes WC, Pfefferbaum A, Schulte T (2013) Midbrain-driven emotion and reward processing in alcoholism. Neuropsychopharmacology 38:1844–1853 23615665 10.1038/npp.2013.102 PMC3746685

[CR68] Muller-Oehring EM, Jung YC, Pfefferbaum A, Sullivan EV, Schulte T (2015) The resting brain of alcoholics. Cereb Cortex 25:4155–4168 24935777 10.1093/cercor/bhu134 PMC4816777

[CR70] Nelson EE, Guyer AE (2011) The development of the ventral prefrontal cortex and social flexibility. Dev Cogn Neurosci 1:233–245 21804907 10.1016/j.dcn.2011.01.002 PMC3143481

[CR71] O’Daly OG, Trick L, Scaife J, Marshall J, Ball D, Phillips ML, Williams SS, Stephens DN, Duka T (2012) Withdrawal-associated increases and decreases in functional neural connectivity associated with altered emotional regulation in alcoholism. Neuropsychopharmacology 37:2267–2276 22617355 10.1038/npp.2012.77 PMC3422491

[CR72] Oberlin BG, Dzemidzic M, Bragulat V, Lehigh CA, Talavage T, O’Connor SJ, Kareken DA (2012) Limbic responses to reward cues correlate with antisocial trait density in heavy drinkers. NeuroImage 60:644–652 22227139 10.1016/j.neuroimage.2011.12.043 PMC3288676

[CR73] Ochsner KN, Bunge SA, Gross JJ, Gabrieli JD (2002) Rethinking feelings: an FMRI study of the cognitive regulation of emotion. J Cogn Neurosci 14:1215–1229 12495527 10.1162/089892902760807212

[CR74] Orr JM, Paschall CJ, Banich MT (2016) Recreational marijuana use impacts white matter integrity and subcortical (but not cortical) morphometry. Neuroimage Clin 12:47–56 27408790 10.1016/j.nicl.2016.06.006 PMC4925620

[CR75] Palomero-Gallagher N, Amunts K (2022) A short review on emotion processing: a lateralized network of neuronal networks. Brain Struct Funct 227:673–684 34216271 10.1007/s00429-021-02331-7 PMC8844151

[CR76] Philippot P, Kornreich C, Blairy S, Baert I, Dulk D, Le Bon A, Streel O, Hess E, Pelc U, Verbanck I, P (1999) Alcoholics’ deficits in the decoding of emotional facial expression. Alcohol Clin Exp Res 23:1031–1038 10397287

[CR77] Porta-Casteras D, Fullana MA, Tinoco D, Martinez-Zalacain I, Pujol J, Palao DJ, Soriano-Mas C, Harrison BJ, Via E, Cardoner N (2020) Prefrontal-amygdala connectivity in trait anxiety and generalized anxiety disorder: testing the boundaries between healthy and pathological worries. J Affect Disord 267:211–219 32217221 10.1016/j.jad.2020.02.029

[CR78] Power JD, Barnes KA, Snyder AZ, Schlaggar BL, Petersen SE (2012) Spurious but systematic correlations in functional connectivity MRI networks arise from subject motion. NeuroImage 59:2142–2154 22019881 10.1016/j.neuroimage.2011.10.018 PMC3254728

[CR79] Puce A, Allison T, Gore JC, McCarthy G (1995) Face-sensitive regions in human extrastriate cortex studied by functional MRI. J Neurophysiol 74:1192–1199 7500143 10.1152/jn.1995.74.3.1192

[CR80] Quirk GJ, Beer JS (2006) Prefrontal involvement in the regulation of emotion: convergence of rat and human studies. Curr Opin Neurobiol 16:723–727 17084617 10.1016/j.conb.2006.07.004

[CR81] Radoman M, Phan KL, Ajilore OA, Gorka SM (2024) Altered effective connectivity during threat anticipation in individuals with alcohol use disorder. Biol Psychiatry Cogn Neurosci Neuroimaging 10.1016/j.bpsc.2024.07.023 PMC11868811 39117274

[CR82] Rolls ET, Huang CC, Lin CP, Feng J, Joliot M (2020) Automated anatomical labelling atlas 3. NeuroImage 206:116189 31521825 10.1016/j.neuroimage.2019.116189

[CR83] Salloum JB, Ramchandani VA, Bodurka J, Rawlings R, Momenan R, George D, Hommer DW (2007) Blunted rostral anterior cingulate response during a simplified decoding task of negative emotional facial expressions in alcoholic patients. Alcohol Clin Exp Res 31:1490–1504 17624997 10.1111/j.1530-0277.2007.00447.x

[CR84] Seghier ML, Zeidman P, Neufeld NH, Leff AP, Price CJ (2010) Identifying abnormal connectivity in patients using dynamic causal modeling of FMRI responses. Front Syst Neurosci 4, PMID: 20838471 10.3389/fnsys.2010.00142 PMC2936900 20838471

[CR85] Seo D, Lacadie CM, Tuit K, Hong KI, Constable RT, Sinha R (2013) Disrupted ventromedial prefrontal function, alcohol craving, and subsequent relapse risk. JAMA Psychiatry 70:727–739 23636842 10.1001/jamapsychiatry.2013.762 PMC3788824

[CR86] Seo D, Martins JS, Sinha R (2024) Brain correlates and functional connectivity linking stress, autonomic dysregulation, and alcohol motivation. Neurobiol Stress 31:100645 38933283 10.1016/j.ynstr.2024.100645 PMC11201348

[CR87] Sergerie K, Chochol C, Armony JL (2008) The role of the amygdala in emotional processing: a quantitative meta-analysis of functional neuroimaging studies. Neurosci Biobehav Rev 32:811–830 18316124 10.1016/j.neubiorev.2007.12.002

[CR88] Sheng F, Wang Y, Li R, Li X, Chen X, Zhang Z, Liu R, Zhang L, Zhou Y, Wang G (2024) Altered effective connectivity among face-processing systems in major depressive disorder. J Psychiatry Neurosci 49:E145–E156 38692692 10.1503/jpn.230123 PMC11068425

[CR89] Silvers JA, Insel C, Powers A, Franz P, Helion C, Martin RE, Weber J, Mischel W, Casey BJ, Ochsner KN 2017. vlPFC-vmPFC-Amygdala interactions underlie Age-Related differences in cognitive regulation of emotion. Cereb Cortex 27, 3502–3514 10.1093/cercor/bhw073 PMC6059245 27341851

[CR90] Sinha R (2022) Alcohol’s negative emotional side: the role of stress neurobiology in alcohol use disorder. Alcohol Res 42:12 36338609 10.35946/arcr.v42.1.12 PMC9621746

[CR91] Snyder AD, Ma L, Steinberg JL, Woisard K, Moeller FG (2021) Dynamic causal modeling self-connectivity findings in the functional magnetic resonance imaging neuropsychiatric literature. Front Neurosci 15:636273 34456665 10.3389/fnins.2021.636273 PMC8385130

[CR92] Song Z, Chen J, Wen Z, Zhang L (2021) Abnormal functional connectivity and effective connectivity between the default mode network and attention networks in patients with alcohol-use disorder. Acta Radiol 62:251–259 32423229 10.1177/0284185120923270

[CR93] Sripada CS, Angstadt M, McNamara P, King AC, Phan KL (2011) Effects of alcohol on brain responses to social signals of threat in humans. NeuroImage 55:371–380 21122818 10.1016/j.neuroimage.2010.11.062 PMC3031714

[CR110] statements & declarations

[CR94] Steimer T (2002) The biology of fear- and anxiety-related behaviors. Dialogues Clin Neurosci 4:231–249 22033741 10.31887/DCNS.2002.4.3/tsteimer PMC3181681

[CR95] Stephan KE, Penny WD, Moran RJ, den Ouden HE, Daunizeau J, Friston KJ (2010) Ten simple rules for dynamic causal modeling. NeuroImage 49:3099–3109 PMID: 19914382 19914382 10.1016/j.neuroimage.2009.11.015 PMC2825373

[CR96] Stoddard J, Tseng WL, Kim P, Chen G, Yi J, Donahue L, Brotman MA, Towbin KE, Pine DS, Leibenluft E (2017) Association of irritability and anxiety with the neural mechanisms of implicit face emotion processing in youths with psychopathology. JAMA Psychiatry 74:95–103 27902832 10.1001/jamapsychiatry.2016.3282 PMC6309540

[CR97] Stout DM, Harle KM, Norman SB, Simmons AN, Spadoni AD (2023) Resting-state connectivity subtype of comorbid PTSD and alcohol use disorder moderates improvement from integrated prolonged exposure therapy in veterans. Psychol Med 53:332–341 33926595 10.1017/S0033291721001513 PMC10880798

[CR98] Suzuki S, Mell MM, O’Malley SS, Krystal JH, Anticevic A, Kober H (2020) Regulation of craving and negative emotion in alcohol use disorder. Biol Psychiatry Cogn Neurosci Neuroimaging 5:239–250 31892465 10.1016/j.bpsc.2019.10.005 PMC7010564

[CR99] Tan HY, Callicott JH, Weinberger DR (2007) Dysfunctional and compensatory prefrontal cortical systems, genes and the pathogenesis of schizophrenia. Cereb Cortex 17(Suppl 1):i171–181 17726000 10.1093/cercor/bhm069

[CR100] Thayer JF, Ahs F, Fredrikson M, Sollers JJ 3rd, Wager TD (2012) A meta-analysis of heart rate variability and neuroimaging studies: implications for heart rate variability as a marker of stress and health. Neurosci Biobehav Rev 36:747–756 22178086 10.1016/j.neubiorev.2011.11.009

[CR102] Van Essen DC, Ugurbil K, Auerbach E, Barch D, Behrens TE, Bucholz R, Chang A, Chen L, Corbetta M, Curtiss SW, Della Penna S, Feinberg D, Glasser MF, Harel N, Heath AC, Larson-Prior L, Marcus D, Michalareas G, Moeller S, Oostenveld R, Petersen SE, Prior F, Schlaggar BL, Smith SM, Snyder AZ, Xu J, Yacoub E (2012) The human connectome project: a data acquisition perspective. NeuroImage 62:2222–2231 22366334 10.1016/j.neuroimage.2012.02.018 PMC3606888

[CR101] Van Essen DC, Smith SM, Barch DM, Behrens TE, Yacoub E, Ugurbil K, Consortium WU-MH (2013) The WU-Minn human connectome project: an overview. NeuroImage 80:62–79 23684880 10.1016/j.neuroimage.2013.05.041 PMC3724347

[CR103] Van Overwalle F, Van de Steen F, Marien P (2019) Dynamic causal modeling of the effective connectivity between the cerebrum and cerebellum in social mentalizing across five studies. Cogn Affect Behav Neurosci 19:211–223 30361864 10.3758/s13415-018-00659-y

[CR104] Voon V, Grodin E, Mandali A, Morris L, Donamayor N, Weidacker K, Kwako L, Goldman D, Koob GF, Momenan R (2020) Addictions neuroimaging assessment (ANIA): towards an integrative framework for alcohol use disorder. Neurosci Biobehav Rev 113:492–506 32298710 10.1016/j.neubiorev.2020.04.004

[CR105] Wang Y, Guo N, Zhao L, Huang H, Yao X, Sang N, Hou X, Mao Y, Bi T, Qiu J (2017) The structural and functional correlates of the efficiency in fearful face detection. Neuropsychologia 100:1–9 28391034 10.1016/j.neuropsychologia.2017.04.004

[CR106] Williams LM (2016) Precision psychiatry: a neural circuit taxonomy for depression and anxiety. Lancet Psychiatry 3:472–480 27150382 10.1016/S2215-0366(15)00579-9 PMC4922884

[CR107] Willinger D, Karipidis II, Haberling I, Berger G, Walitza S, Brem S (2022) Deficient prefrontal-amygdalar connectivity underlies inefficient face processing in adolescent major depressive disorder. Transl Psychiatry 12:195 35538052 10.1038/s41398-022-01955-5 PMC9090758

[CR108] Wudarczyk OA, Kohn N, Bergs R, Goerlich KS, Gur RE, Turetsky B, Schneider F, Habel U (2016) Chemosensory anxiety cues enhance the perception of fearful faces - An fMRI study. NeuroImage 143:214–222 27592811 10.1016/j.neuroimage.2016.09.002

[CR109] Xia M, Wang J, He Y (2013) BrainNet viewer: a network visualization tool for human brain connectomics. PLoS ONE 8, e68910 10.1371/journal.pone.0068910 PMC3701683 23861951

