# Supplementary material for: Altered effective connectivity of emotion perception and regulation networks during an emotional face perception task in adults with alcohol use disorder
Source: Brain Struct Funct. 2025 Aug 14;230(7):136. doi: 10.1007/s00429-025-02992-8 (PMC12354520; doi:10.1007/s00429-025-02992-8)
Supplement: Supplementary file 1 — Supplementary Material 1 [file 429_2025_2992_MOESM1_ESM.docx]

**Supplementary Online Content**

Hammond CJ*, Ma* L, Bjork J, Moeller FG, Arias AJ. Altered effective connectivity of emotion perception and regulation networks during an emotional face perception task in adults with alcohol use disorder. *Brain Structure and Function*.

**Corresponding Author**: Christopher J. Hammond, M.D., Ph.D. Email: [chammo20@jhmi.edu](mailto:chammo20@jhmi.edu)

**Table of Contents:**

**Supplementary Table S1.** Items and corresponding HCP variables and priority weights used to select the 70 Control participants from the remaining participants (after applying the exclusion criteria and inclusion criteria for the Alcohol Use Disorder group) to match the 70 AUD participants. The selection procedure was implemented by an in-house Matlab code. For each Alcohol participant, the matching Control participant was selected by minimizing summation of the weighted (i.e., times the priority weight) group differences for all the items. Each selected participant was marked in order to avoid repeated selection.

**Supplementary Table S2.** The MNI coordinates of the DCM nodes and the re-positioned DCM nodes.

**Supplementary Table S3.** The brain activation clusters for emotional-face minus neutral-shape contrast in combined sample (n=140) including AUD and CON groups, used to constrain the a priori-selected DCM nodes.

**Supplemental Results.** Results from supplemental DCM analyses examining sex differences and post-hoc linear regression analyses examining associations between ECs and alcohol usage, anxiety, and depression separately in AUD and CON groups.

**Supplementary Table S4.** The results of the DCM PEB analyses testing the interaction between Group (AUD vs. Con) and Sex (Male vs. Female).

**Supplementary Table S5.** The results of the DCM PEB analyses testing the interaction between Group (AUD vs. Con) and Birth control status (with birth control vs. without birth control) in all female participants.

**Supplementary Table S6.** The results of the linear regression analyses examining associations between ECs derived from DCM PEB analyses and alcohol usage, anxiety, and depression in the AUD participant group.

**Supplementary Table S7.** The results of the linear regression analyses examining associations between ECs derived from DCM PEB analyses and alcohol usage, anxiety, and depression in the CON participant group.

**-------------------------------------------------------------------------------------------------------------------------------**

Supplementary Table S1: Items and corresponding HCP variables and priority weights used to select the 101 Control participants from the 638 participants (after applying the exclusion criteria and inclusion criteria for the Alcohol Use Disorder group) to match the 101 Alcohol participants. The selection procedure was implemented by an in-house Matlab code. For each Alcohol participant, the matching Control participant was selected by minimizing summation of the weighted (i.e., times the priority weight) group differences for all the items. Each selected participant was marked in order to avoid repeated selection.

| **Matching item** | **HCP variable** | **Priority weight** |
| --- | --- | --- |
| 1. Sex | Gender | 2 |
| 2. Age | Age_in_Yrs | 1 |
| 3. Education | SSAGA_Educ | 1 |
| 4. Ever used marijuana? | SSAGA_Mj_Use | 1 |
| 5. Times used marijuana | SSAGA_Mj_Times_Used | 1 |
| 6. Number days smoked/used ANY TOBACCO in past 7 days | Total_Any_Tobacco_7days | 2 |
| 7. Smoking history | SSAGA_TB_Smoking_History | 2 |
| 8. Still smoking? | SSAGA_TB_Still_Smoking | 2 |
| 9. Fagerstrom FTND score | SSAGA_FTND_Score | 2 |
| 10. Fagerstrom HSI score | SSAGA_HSI_Score | 2 |

**Supplementary Table S2:** The MNI coordinates of the DCM nodes and the re-positioned DCM nodes.

| Node name | MNI coordinates of the initial DCM nodes | MNI coordinates of the re-positioned DCM nodes (mean ± STD) |
| --- | --- | --- |
| VMPFC | x: 2  y: 58  z: ‒8 | x: 2.22 ± 6.75  y: 58.01 ± 6.98  z: ‒7.80 ± 6.32 |
| Left VLPFC | x: ‒44  y: 18  z: 26 | x: ‒44.83 ± 6.75  y: 17.50 ± 7.42  z: 26.10 ± 6.81 |
| Right VLPFC | x: 52  y: 18  z: 30 | x: 50.39 ± 4.77  y: 16.54 ± 7.11  z: 29.78 ± 6.61 |
| Left amygdala | x: ‒20  y: ‒2  z: ‒16 | x: ‒20.39 ± 6.46  y: ‒2.42 ± 6.55  z: ‒15.52 ± 5.52 |
| Right amygdala | x: 20  y: ‒2  z: ‒14 | x: 20.63 ± 6.60  y: ‒1.88 ± 6.48  z: ‒14.31 ± 5.74 |
| Left FG | x: ‒40  y: ‒52  z: ‒20 | x: ‒40.32 ± 5.91  y: ‒56.83 ± 6.30  z: ‒20.27 ± 5.28 |
| Right FG | x: 42  y: ‒52  z: ‒18 | x: 44.59 ± 5.60  y: ‒55.08 ± 6.27  z: ‒16.77 ± 5.18 |
| Hypothalamus | x: 4  y: ‒6  z: ‒12 | x: 4.49 ± 3.37  y: ‒6.12 ± 3.26  z: ‒12.07 ± 3.34 |

**Supplementary Table S3.** The brain activation clusters detected by SPM12 second-level random effects 1-sample t test analysis for the contrast of emotional-face minus neutral-shape when the two groups were combined (n=140), with cluster-defining threshold p=0.001 and cluster level p < 0.05 (FWE corrected, two-tail). These brain activations were used to constrain the a priori-selected DCM nodes. x, y, and z = MNI standard space coordinates (mm). Negative x = Left hemisphere. L=left. R=right.

| Anatomical description | AAL3 Label Number | Number of voxels | Maximal t value within labeled region | MNI coordinates [x y z] (mm) of voxel with maximal t | |
| --- | --- | --- | --- | --- | --- |
| L Precentral gyrus | 1 | 318 | 10.60 | -42, 12, 30 | |
| R Precentral gyrus | 2 | 379 | 9.24 | 48, 10, 38 | |
| L Superior frontal gyrus, dorsolateral | 3 | 317 | 8.12 | -14, 46, 44 | |
| R Superior frontal gyrus, dorsolateral | 4 | 353 | 6.70 | 16, 38, 50 | |
| L Middle frontal gyrus | 5 | 467 | 9.15 | -46, 46, -2 | |
| R Middle frontal gyrus | 6 | 604 | 12.21 | 44, 28, 22 | |
| L Inferior frontal gyrus, opercular part | 7 | 274 | 11.56 | -40, 12, 28 | |
| R Inferior frontal gyrus, opercular part | 8 | 459 | 15.07 | 52, 18, 30 | |
| L Inferior frontal gyrus, triangular part | 9 | 1212 | 14.39 | -46, 16, 26 | |
| R Inferior frontal gyrus, triangular part | 10 | 886 | 14.32 | 48, 20, 28 | |
| L IFG pars orbitalis | 11 | 351 | 9.80 | -40, 28, -12 | |
| R IFG pars orbitalis | 12 | 203 | 7.91 | 38, 36, -10 | |
| L Supplementary motor area | 15 | 125 | 6.40 | -2, 24, 54 | |
| R Supplementary motor area | 16 | 134 | 5.64 | 4, 16, 54 | |
| L Superior frontal gyrus, medial | 19 | 626 | 10.35 | 0, 40, 44 | |
| R Superior frontal gyrus, medial | 20 | 598 | 8.65 | 4, 42, 50 | |
| L Superior frontal gyrus, medial orbital | 21 | 31 | 7.24 | -4, 54, -10 | |
| R Superior frontal gyrus, medial orbital | 22 | 6 | 6.52 | 2, 54, -8 | |
| R Anterior orbital gyrus | 28 | 1 | 6.63 | 42, 36, -14 | |
| L Posterior orbital gyrus | 29 | 12 | 10.82 | -38, 32, -14 | |
| R Posterior orbital gyrus | 30 | 5 | 7.13 | 40, 32, -14 | |
| L Lateral orbital gyrus | 31 | 8 | 10.79 | -38, 34, -14 |  |
| R Lateral orbital gyrus | 32 | 4 | 5.55 | 44, 36, -14 |  |
| L Insula | 33 | 103 | 7.83 | -30, 12, -20 |  |
| R Insula | 34 | 15 | 5.41 | 30, 10, -16 |  |
| R Middle cingulate & paracingulate gyri | 38 | 30 | 6.98 | 6, 4, 30 |  |
| L Posterior cingulate gyrus | 39 | 1 | 3.55 | 2, -52, 28 |  |
| R Posterior cingulate gyrus | 40 | 6 | 5.51 | 4, -58, 30 |  |
| L Hippocampus | 41 | 363 | 15.93 | -18, -6, -14 |  |
| R Hippocampus | 42 | 424 | 17.05 | 20, -4, -14 |  |
| L Parahippocampal gyrus | 43 | 7 | 4.81 | -14, -30, -10 |  |
| R Parahippocampal gyrus | 44 | 68 | 12.18 | 18, -2, -18 |  |
| L Amygdala | 45 | 122 | 16.04 | -20, -2, -16 |  |
| R Amygdala | 46 | 165 | 17.64 | 20, -2, -14 |  |
| L Calcarine fissure and surrounding cortex | 47 | 569 | 18.04 | -14, -94, -10 |  |
| R Calcarine fissure and surrounding cortex | 48 | 513 | 19.56 | 18, -96, -6 |  |
| L Cuneus | 49 | 52 | 5.19 | 2, -72, 26 |  |
| R Cuneus | 50 | 185 | 5.85 | 24, -64, 22 |  |
| L Lingual gyrus | 51 | 501 | 16.27 | -12, -94, -12 |  |
| R Lingual gyrus | 52 | 431 | 22.24 | 22, -92, -10 |  |
| L Superior occipital gyrus | 53 | 20 | 6.18 | -18, -92, 2 |  |
| R Superior occipital gyrus | 54 | 48 | 8.27 | 24, -86, 2 |  |
| L Middle occipital gyrus | 55 | 438 | 21.69 | -22, -96, -4 |  |
| R Middle occipital gyrus | 56 | 113 | 13.34 | 26, -88, 0 |  |
| L Inferior occipital gyrus | 57 | 392 | 22.74 | -24, -96, -4 |  |
| R Inferior occipital gyrus | 58 | 152 | 20.97 | 30, -90, -8 |  |
| L Fusiform gyrus | 59 | 371 | 19.09 | -38, -50, -20 |  |
| R Fusiform gyrus | 60 | 224 | 21.44 | 40, -54, -18 |  |
| L Postcentral gyrus | 61 | 204 | 9.24 | -18, -28, 72 |  |
| R Postcentral gyrus | 62 | 18 | 6.97 | 42, -20, 50 |  |
| L Superior parietal gyrus | 63 | 21 | 4.95 | -24, -40, 64 |  |
| R Superior parietal gyrus | 64 | 1 | 3.30 | 32, -56, 48 |  |
| L Inferior parietal gyrus, excluding supramarginal and angular gyri | 65 | 90 | 4.85 | -28, -74, 46 |  |
| R Inferior parietal gyrus, excluding supramarginal and angular gyri | 66 | 63 | 6.40 | 38, -56, 42 |  |
| L SupraMarginal gyrus | 67 | 2 | 3.67 | -48, -50, 24 |  |
| L Angular gyrus | 69 | 225 | 9.07 | -46, -68, 24 |  |
| R Angular gyrus | 70 | 208 | 7.68 | 46, -64, 24 |  |
| L Precuneus | 71 | 252 | 7.37 | -10, -44, 66 |  |
| R Precuneus | 72 | 377 | 7.80 | 2, -60, 40 |  |
| L Paracentral lobule | 73 | 56 | 10.24 | -16, -28, 72 |  |
| R Paracentral lobule | 74 | 32 | 5.61 | 10, -42, 68 |  |
| L Caudate nucleus | 75 | 233 | 8.86 | -6, 16, 8 |  |
| R Caudate nucleus | 76 | 101 | 6.96 | 6, 18, 6 |  |
| L Lenticular nucleus, Putamen | 77 | 7 | 4.62 | -22, 4, -8 |  |
| R Lenticular nucleus, Putamen | 78 | 13 | 4.20 | 32, 2, -10 |  |
| L Superior temporal gyrus | 85 | 95 | 6.90 | -54, -48, 16 |  |
| R Superior temporal gyrus | 86 | 386 | 10.37 | 48, -40, 12 |  |
| L Temporal pole: superior temporal gyrus | 87 | 119 | 13.15 | -32, 6, -24 |  |
| R Temporal pole: superior temporal gyrus | 88 | 151 | 12.13 | 36, 6, -20 |  |
| L Middle temporal gyrus | 89 | 990 | 10.10 | -46, -68, 22 |  |
| R Middle temporal gyrus | 90 | 466 | 9.87 | 56, -2, -16 |  |
| L Temporal pole: middle temporal gyrus | 91 | 41 | 7.36 | -38, 8, -28 |  |
| R Temporal pole: middle temporal gyrus | 92 | 50 | 8.14 | 48, 14, -28 |  |
| L Inferior temporal gyrus | 93 | 43 | 9.72 | -40, -46, -14 |  |
| R Inferior temporal gyrus | 94 | 41 | 12.33 | 42, -60, -12 |  |
| L Crus I of cerebellar hemisphere | 95 | 105 | 8.69 | -4, -74, -26 |  |
| R Crus I of cerebellar hemisphere | 96 | 11 | 7.44 | 8, -76, -24 |  |
| L Crus II of cerebellar | 97 | 11 | 8.02 | -4, -78, -26 |  |
| R Lobule III of cerebellar hemisphere | 100 | 2 | 3.23 | 8, -42, -12 |  |
| L Lobule IV, V of cerebellar hemisphere | 101 | 38 | 4.94 | -8, -38, -4 |  |
| R Lobule IV, V of cerebellar hemisphere | 102 | 27 | 6.63 | 30, -42, -22 |  |
| L Lobule VI of cerebellar hemisphere | 103 | 22 | 6.58 | -32, -48, -24 |  |
| R Lobule VI of cerebellar hemisphere | 104 | 19 | 6.66 | 8, -76, -22 |  |
| L Lobule VIII of cerebellar hemisphere | 107 | 3 | 4.04 | -4, -60, -38 |  |
| L Lobule IX of cerebellar hemisphere | 109 | 123 | 6.46 | -4, -48, -34 |  |
| R Lobule IX of cerebellar hemisphere | 110 | 66 | 4.77 | 2, -60, -46 |  |
| Lobule III of vermis | 114 | 25 | 4.28 | 4, -36, -4 |  |
| Lobule IV, V of vermis | 115 | 5 | 4.55 | -4, -64, -2 |  |
| Lobule VI of vermis | 116 | 7 | 4.64 | 6, -76, -20 |  |
| Lobule VII of vermis | 117 | 39 | 10.11 | -2, -74, -28 |  |
| Lobule VIII of vermis | 118 | 3 | 3.40 | -2, -62, -38 |  |
| Lobule IX of vermis | 119 | 105 | 5.89 | 0, -52, -36 |  |
| Lobule X of vermis | 120 | 40 | 6.74 | -4, -48, -32 |  |
| R Thalamus, Lateral posterior | 124 | 9 | 4.81 | 10, -16, 14 |  |
| R Thalamus, Ventral lateral | 128 | 31 | 4.99 | 12, -16, 14 |  |
| R Thalamus, Ventral posterolateral | 130 | 16 | 4.54 | 14, -18, 14 |  |
| R Thalamus, Mediodorsal medial magnocellular | 136 | 10 | 4.03 | 2, -18, 6 |  |
| R Thalamus, Mediodorsal lateral parvocellular | 138 | 6 | 3.67 | 8, -14, 10 |  |
| L Thalamus, Lateral geniculate | 139 | 13 | 4.70 | -18, -30, -2 |  |
| R Thalamus, Lateral geniculate | 140 | 14 | 7.44 | 18, -30, -2 |  |
| L Thalamus, Medial Geniculate | 141 | 3 | 3.24 | -18, -26, -4 |  |
| R Thalamus, Medial Geniculate | 142 | 8 | 5.97 | 16, -24, -8 |  |
| L Thalamus, Pulvinar anterior | 143 | 12 | 6.39 | -12, -30, -2 |  |
| R Thalamus, Pulvinar anterior | 144 | 21 | 9.36 | 16, -32, -2 |  |
| L Thalamus, Pulvinar medial | 145 | 20 | 7.73 | -12, -32, 0 |  |
| R Thalamus, Pulvinar medial | 146 | 69 | 8.74 | 10, -30, 0 |  |
| R Thalamus, Pulvinar lateral | 148 | 1 | 3.47 | 12, -26, 0 |  |
| R Thalamus, Pulvinar inferior | 150 | 1 | 3.43 | 12, -18, 16 |  |
| R Anterior cingulate cortex, pregenual | 154 | 2 | 3.96 | 4, 48, 28 |  |
| L Anterior cingulate cortex, supracallosal | 155 | 22 | 6.55 | 0, 4, 28 |  |
| R Anterior cingulate cortex, supracallosal | 156 | 30 | 7.66 | 4, 6, 28 |  |
| L Nucleus accumbens | 157 | 4 | 3.71 | -6, 14, -2 |  |
| R Nucleus accumbens | 158 | 1 | 3.44 | 4, 14, -2 |  |

**Supplemental Results:**

**Results of Supplemental DCM analyses testing for sex and group-by-sex interaction effects.**

For this analysis we conducted a supplementary DCM group-level analysis testing for sex differences between male and female participants and testing the interaction between Group (70 participants with AUD vs. 70 healthy controls) and Sex (69 Males vs. 71 Females). This analysis revealed Sex differences in multiple ECs and Group X Sex interactions for some ECs (see supplemental Table S4).

Given the widespread sex differences and group-by-sex interaction effects observed, we examined factors that might contribute to sex and group-by-sex differences in ECs. HCP collected several measures related to menstrual history in female participants, and for our post-hoc analysis we focused on two specific measures (1) birth control status (i.e., current use of hormonal or fertility-related medication) and (2) days since last menstrual period as these measures were available in most female participants in our sample and were deemed the most appropriate for controlling effects related to menstrual cycle or hormonal fluctuations. Regarding Birth Control effects, we conducted a supplementary analysis to test for a modulating effect of Birth Control Status on EC in female participants in the sample (32 using vs. 39 not using birth control), and also examine the interaction between Group (33 female participants with AUD vs. 38 healthy controls) and Birth Control Status. Results of these analyses showed that Birth Control Status modulated certain ECs in female participants and identified significant/reliable Group × Birth Control Status interactions for some ECs (see supplemental Table S5).

Regarding days since last menstrual period, for this measure we noted some extremely large values (e.g., 1539 days), even among participants not using birth control. To assess the potential effects of menstrual circle, we performed a supplementary linear regression analysis testing the association between each EC and days since last menstrual period in all female participants (n = 71), and a second analysis restricted to participants with fewer than 30 days since last period (n = 54). Neither analysis showed a significant association (beta = 0, PP = 0 for all ECs).

**Results of Linear regression analyses examining associations between EC and cumulative alcohol usage, anxiety and depression scores separately for AUD and CON groups.**

For this analysis we ran each linear regression analysis (EC in relation to cumulative alcohol usage, depression scores, and anxiety scores) separately in AUD and CON groups (shown below in Tables S6 and S7). No significant linear relationship was observed between any of the EC measures and alcohol usage or anxiety scores in either the AUD or CON group (all betas = 0, PP = 0 for all ECs). For depression, no ECs were associated with depression scores in the CON group (all betas = 0, PP = 0 for all ECs). In the AUD group, 61 out of 64 ECs showed no relationship with depression scores (all betas = 0, PP = 0), while three ECs showed potential associations: R amygdala to L FG EC (beta = –0.0221, PP = 0.9479), R amygdala to hypothalamus EC (beta = 0.0287, PP = 0.9858), and R FG to R amygdala EC (beta = –0.0090, PP = 0.5248). A Wald-type Z-test was used to compare group differences in regression coefficients based on beta values and their standard errors. Significant group differences were found for R amygdala to L FG EC (Z = –2.9104, p = 0.0036) and R amygdala to hypothalamus EC (Z = 3.4692, p = 0.0005). The group difference for R FG to R amygdala EC was not significant (Z = –0.9016, p = 0.3673).

**Supplementary Table S4.** The results of the DCM PEB analyses testing the interaction between Group (AUD vs. Con) and Sex (Male vs. Female). For each node-to-node connection in the each of the group level PEB analyses, an EC magnitude (absolute value in Hz) and Bayesian posterior probabilities (PP) between 0 and 1 (0≤PP≤1) is shown. For interpretation purposes: Larger magnitude ECs with Bayesian-PP ≥0.95 are considered reliable. Test-retest reliability of ECs with larger magnitudes is higher. Bayesian-PP is the conditional probability computed by PEB using Bayes rules after likelihood function and prior probability density of model parameters are taken into account. The higher the Bayesian-PP, the greater confidence, although the concept of statistical significance does not directly apply. DCM experts recommend focusing on EC results with PP ≥ 0.90 or 0.95 as they reflect the most probable effects. In this report, we considered an EC finding reliable if Bayesian-PP ≥0.95 (corresponding to a Bayes-factor of 20). Abbreviations: EC=effective connectivity, PP=posterior probability, L=left, and R=right. VMPFC = ventromedial prefrontal cortex, VLPFC = ventrolateral prefrontal cortex, AMY = amygdala, FG = fusform gyrus, HTH = hypothalamus.

|  | EC in all participants (n=140) | | **Group difference:**  AUD (n=70) minus CON (n=70) | | **Sex difference:**  Male (n=69) minus Female (n=71) | | **Interaction between Group and Sex** | |
| --- | --- | --- | --- | --- | --- | --- | --- | --- |
| **Connectivity** | **EC (Hz)** | **PP** | **EC (Hz)** | **PP** | **beta** | **PP** | **beta** | **PP** |
| VMPFC → VMPFC | 0.1754 | 1 | 0.2802 | 1 | 0.0000 | 0 | 0.0000 | 0 |
| VMPFC → L VLPFC | 0.0000 | 0 | -0.1179 | 1 | -0.1020 | 1 | 0.1396 | 1 |
| VMPFC → R VLPFC | -0.0885 | 1 | -0.0923 | 1 | -0.1557 | 1 | 0.0815 | 1 |
| VMPFC → L AMY | 0.1207 | 1 | 0.0000 | 0 | 0.0000 | 0 | -0.1163 | 1 |
| VMPFC → R AMY | 0.0000 | 0 | -0.0724 | 1 | 0.0000 | 0 | -0.0624 | 1 |
| VMPFC → L FG | 0.0000 | 0 | -0.0919 | 1 | 0.0000 | 0 | 0.0600 | 1 |
| VMPFC → R FG | 0.0000 | 0 | 0.0000 | 0 | -0.1284 | 1 | 0.0000 | 0 |
| VMPFC → HTH | 0.0000 | 0 | 0.0000 | 0 | -0.1476 | 1 | 0.0000 | 0 |
| L VLPFC → VMPFC | -0.1214 | 1 | -0.1251 | 1 | -0.1360 | 1 | 0.0000 | 0 |
| L VLPFC → L VLPFC | 0.5013 | 1 | -0.2044 | 1 | 0.2556 | 1 | -0.1963 | 1 |
| L VLPFC → R VLPFC | -0.2288 | 1 | 0.0953 | 1 | 0.0000 | 0 | 0.0661 | 1 |
| L VLPFC → L AMY | -0.2197 | 1 | 0.0000 | 0 | 0.0000 | 0 | -0.0703 | 1 |
| L VLPFC → R AMY | -0.2623 | 1 | 0.0000 | 0 | -0.0751 | 1 | -0.2412 | 1 |
| L VLPFC → L FG | -0.2191 | 1 | 0.1160 | 1 | 0.0000 | 0 | 0.0714 | 1 |
| L VLPFC → R FG | -0.1804 | 1 | 0.0851 | 1 | -0.0719 | 1 | -0.0301 | 0.537356659 |
| L VLPFC → HTH | -0.2380 | 1 | -0.0905 | 1 | 0.2746 | 1 | 0.0000 | 0 |
| R VLPFC → VMPFC | 0.0000 | 0 | 0.0000 | 0 | -0.0980 | 1 | 0.0000 | 0 |
| R VLPFC → L VLPFC | 0.1971 | 1 | 0.0000 | 0 | 0.2246 | 1 | 0.0000 | 0 |
| R VLPFC → R VLPFC | -0.4312 | 1 | 0.0000 | 0 | -0.2990 | 1 | 0.0000 | 0 |
| R VLPFC → L AMY | 0.1371 | 1 | 0.0845 | 1 | -0.0503 | 0.795541603 | -0.1757 | 1 |
| R VLPFC → R AMY | 0.1278 | 1 | 0.0000 | 0 | 0.2193 | 1 | 0.1151 | 1 |
| R VLPFC → L FG | 0.1704 | 1 | -0.0851 | 1 | 0.1767 | 1 | 0.0000 | 0 |
| R VLPFC → R FG | 0.0717 | 1 | 0.0000 | 0 | 0.1633 | 1 | 0.0743 | 1 |
| R VLPFC → HTH | 0.0000 | 0 | 0.0000 | 0 | 0.0000 | 0 | 0.0000 | 0 |
| L AMY → VMPFC | 0.3894 | 1 | 0.0000 | 0 | -0.0921 | 1 | 0.0000 | 0 |
| L AMY → L VLPFC | 0.2051 | 1 | 0.1553 | 1 | 0.0852 | 1 | -0.2413 | 1 |
| L AMY → R VLPFC | 0.2121 | 1 | 0.0735 | 1 | -0.1180 | 1 | -0.2348 | 1 |
| L AMY → L AMY | 0.1354 | 1 | -0.0533 | 0.641071662 | 0.0000 | 0 | 0.0000 | 0 |
| L AMY → R AMY | 0.2192 | 1 | 0.0000 | 0 | -0.1859 | 1 | 0.0000 | 0 |
| L AMY → L FG | 0.2056 | 1 | 0.0000 | 0 | 0.0976 | 1 | -0.1510 | 1 |
| L AMY → R FG | 0.3203 | 1 | 0.0376 | 0.673764086 | 0.0698 | 1 | -0.1429 | 1 |
| L AMY → HTH | 0.2987 | 1 | 0.2566 | 1 | 0.0000 | 0 | 0.0000 | 0 |
| R AMY → VMPFC | -0.2273 | 1 | 0.1309 | 1 | 0.2255 | 1 | -0.0887 | 1 |
| R AMY → L VLPFC | 0.0000 | 0 | 0.0000 | 0 | -0.2977 | 1 | 0.0888 | 1 |
| R AMY → R VLPFC | 0.0000 | 0 | 0.1518 | 1 | -0.1871 | 1 | 0.3267 | 1 |
| R AMY → L AMY | 0.0868 | 1 | 0.0000 | 0 | -0.1306 | 1 | 0.2622 | 1 |
| R AMY → R AMY | 0.1613 | 1 | 0.0000 | 0 | 0.0000 | 0 | 0.0000 | 0 |
| R AMY → L FG | 0.0000 | 0 | 0.2635 | 1 | -0.3747 | 1 | 0.3271 | 1 |
| R AMY → R FG | -0.2166 | 1 | 0.1030 | 1 | -0.2445 | 1 | 0.2133 | 1 |
| R AMY → HTH | -0.0998 | 1 | 0.0516 | 0.759319463 | -0.0476 | 0.742607855 | -0.0781 | 1 |
| L FG → VMPFC | 0.3515 | 1 | 0.1888 | 1 | 0.1840 | 1 | 0.0000 | 0 |
| L FG → L VLPFC | 0.0000 | 0 | 0.0000 | 0 | 0.2760 | 1 | 0.0328 | 0.596740367 |
| L FG → R VLPFC | 0.1314 | 1 | -0.0969 | 1 | 0.1978 | 1 | 0.0000 | 0 |
| L FG → L AMY | 0.2196 | 1 | 0.0000 | 0 | 0.0000 | 0 | 0.0000 | 0 |
| L FG → R AMY | 0.2455 | 1 | 0.2087 | 1 | -0.0827 | 1 | 0.0910 | 1 |
| L FG → L FG | 0.1637 | 1 | 0.0000 | 0 | -0.1788 | 1 | 0.0000 | 0 |
| L FG → R FG | 0.0629 | 1 | -0.0663 | 1 | 0.0000 | 0 | 0.0000 | 0 |
| L FG → HTH | 0.1454 | 1 | 0.1173 | 1 | 0.2187 | 1 | -0.1194 | 1 |
| R FG → VMPFC | -0.2280 | 1 | -0.1547 | 1 | -0.1339 | 1 | 0.0000 | 0 |
| R FG → L VLPFC | 0.0000 | 0 | -0.0892 | 1 | -0.3053 | 1 | -0.0907 | 1 |
| R FG → R VLPFC | -0.0774 | 1 | 0.0000 | 0 | -0.1613 | 1 | 0.0000 | 0 |
| R FG → L AMY | -0.1555 | 1 | -0.0713 | 1 | 0.0691 | 1 | 0.0745 | 1 |
| R FG → R AMY | -0.2056 | 1 | -0.1336 | 1 | 0.0000 | 0 | 0.0457 | 0.737491496 |
| R FG → L FG | 0.1006 | 1 | -0.0822 | 1 | -0.0862 | 1 | 0.0000 | 0 |
| R FG → R FG | -0.1113 | 1 | 0.0000 | 0 | 0.0000 | 0 | 0.0000 | 0 |
| R FG → HTH | -0.1408 | 1 | -0.2103 | 1 | -0.3918 | 1 | 0.0000 | 0 |
| HTH → VMPFC | -0.0914 | 1 | 0.0724 | 1 | 0.0000 | 0 | 0.0000 | 0 |
| HTH → L VLPFC | -0.1785 | 1 | 0.0000 | 0 | 0.2482 | 1 | 0.0000 | 0 |
| HTH → R VLPFC | -0.2671 | 1 | -0.1237 | 1 | 0.2801 | 1 | -0.1953 | 1 |
| HTH → L AMY | -0.1254 | 1 | 0.0000 | 0 | 0.1631 | 1 | -0.0999 | 1 |
| HTH → R AMY | -0.1112 | 1 | 0.0000 | 0 | 0.0878 | 1 | -0.1239 | 1 |
| HTH → L FG | -0.3873 | 1 | -0.0843 | 1 | 0.1929 | 1 | -0.3298 | 1 |
| HTH → R FG | -0.2491 | 1 | -0.0664 | 1 | 0.2219 | 1 | -0.1844 | 1 |
| HTH → HTH | -0.1448 | 1 | 0.1183 | 1 | 0.0000 | 0 | -0.1501 | 1 |

**Supplementary Table S5.** The results of the DCM PEB analyses testing the interaction between Group (AUD vs. Con) and Birth control status (with birth control vs. without birth control) in all female participants. For each node-to-node connection in the each of the group level PEB analyses, an EC magnitude (absolute value in Hz) and Bayesian posterior probabilities (PP) between 0 and 1 (0≤PP≤1) is shown. For interpretation purposes: Larger magnitude ECs with Bayesian-PP ≥0.95 are considered reliable. Test-retest reliability of ECs with larger magnitudes is higher. Bayesian-PP is the conditional probability computed by PEB using Bayes rules after likelihood function and prior probability density of model parameters are taken into account. The higher the Bayesian-PP, the greater confidence, although the concept of statistical significance does not directly apply. DCM experts recommend focusing on EC results with PP ≥ 0.90 or 0.95 as they reflect the most probable effects. In this report, we considered an EC finding reliable if Bayesian-PP ≥0.95 (corresponding to a Bayes-factor of 20). Abbreviations: EC=effective connectivity, PP=posterior probability, L=left, and R=right. VMPFC = ventromedial prefrontal cortex, VLPFC = ventrolateral prefrontal cortex, AMY = amygdala, FG = fusform gyrus, HTH = hypothalamus.

|  | EC in all female participants (n=71) | | **Group difference:**  AUD (n=33) minus CON (n=38) | | **Difference in birth control:**  With birth control (n=32) minus Without birth control (n=39) | | **Interaction between Group and Birth control** | |
| --- | --- | --- | --- | --- | --- | --- | --- | --- |
| **Connectivity** | **EC (Hz)** | **PP** | **EC (Hz)** | **PP** | **beta** | **PP** | **beta** | **PP** |
| VMPFC → VMPFC | 0.0868 | 0.730324865 | 0.2508 | 1 | 0.1366 | 1 | -0.2048 | 1 |
| VMPFC → L VLPFC | 0.1038 | 1 | 0.0000 | 0 | -0.2174 | 1 | 0.0000 | 0 |
| VMPFC → R VLPFC | 0.0896 | 1 | -0.1193 | 1 | 0.0000 | 0 | 0.0000 | 0 |
| VMPFC → L AMY | 0.2417 | 1 | 0.0899 | 1 | 0.0000 | 0 | -0.1164 | 1 |
| VMPFC → R AMY | 0.0000 | 0 | 0.0000 | 0 | 0.0000 | 0 | 0.0000 | 0 |
| VMPFC → L FG | 0.2077 | 1 | 0.0000 | 0 | -0.1472 | 1 | -0.1792 | 1 |
| VMPFC → R FG | 0.2457 | 1 | 0.1007 | 1 | 0.0000 | 0 | -0.1436 | 1 |
| VMPFC → HTH | 0.1307 | 1 | 0.0806 | 1 | 0.0000 | 0 | 0.0000 | 0 |
| L VLPFC → VMPFC | 0.0000 | 0 | 0.0000 | 0 | 0.0000 | 0 | 0.0000 | 0 |
| L VLPFC → L VLPFC | 0.1489 | 1 | 0.0000 | 0 | 0.0000 | 0 | 0.0000 | 0 |
| L VLPFC → R VLPFC | -0.3047 | 1 | -0.1001 | 1 | 0.1708 | 1 | 0.0000 | 0 |
| L VLPFC → L AMY | -0.1404 | 1 | 0.0000 | 0 | 0.0000 | 0 | 0.0000 | 0 |
| L VLPFC → R AMY | -0.1712 | 1 | 0.2043 | 1 | 0.0000 | 0 | 0.0000 | 0 |
| L VLPFC → L FG | -0.2407 | 1 | 0.0000 | 0 | 0.1365 | 1 | 0.0000 | 0 |
| L VLPFC → R FG | -0.1181 | 1 | 0.0000 | 0 | 0.1316 | 1 | 0.0000 | 0 |
| L VLPFC → HTH | -0.3690 | 1 | 0.0000 | 0 | -0.1309 | 1 | -0.2378 | 1 |
| R VLPFC → VMPFC | 0.0000 | 0 | -0.1097 | 1 | 0.0000 | 0 | 0.0000 | 0 |
| R VLPFC → L VLPFC | -0.1598 | 1 | -0.2139 | 1 | 0.1101 | 1 | 0.0000 | 0 |
| R VLPFC → R VLPFC | -0.1264 | 1 | 0.0000 | 0 | 0.0000 | 0 | 0.0000 | 0 |
| R VLPFC → L AMY | 0.0884 | 1 | 0.2367 | 1 | 0.0000 | 0 | 0.1621 | 1 |
| R VLPFC → R AMY | 0.0000 | 0 | -0.1327 | 1 | 0.0000 | 0 | -0.1469 | 1 |
| R VLPFC → L FG | 0.0000 | 0 | -0.1737 | 1 | 0.2826 | 1 | 0.0000 | 0 |
| R VLPFC → R FG | 0.0000 | 0 | 0.0000 | 0 | 0.0000 | 0 | 0.0982 | 1 |
| R VLPFC → HTH | 0.0000 | 0 | 0.0000 | 0 | 0.0000 | 0 | 0.1362 | 1 |
| L AMY → VMPFC | 0.2003 | 1 | 0.0000 | 0 | 0.1881 | 1 | 0.0000 | 0 |
| L AMY → L VLPFC | 0.0000 | 0 | 0.0000 | 0 | 0.2259 | 1 | 0.1169 | 1 |
| L AMY → R VLPFC | -0.0991 | 1 | 0.1071 | 1 | 0.0884 | 1 | 0.3254 | 1 |
| L AMY → L AMY | 0.1571 | 1 | 0.0000 | 0 | 0.0000 | 0 | 0.2216 | 1 |
| L AMY → R AMY | 0.1369 | 1 | 0.0000 | 0 | 0.0000 | 0 | 0.1440 | 1 |
| L AMY → L FG | -0.2228 | 1 | 0.1707 | 1 | 0.1015 | 1 | 0.3221 | 1 |
| L AMY → R FG | -0.1032 | 1 | 0.0000 | 0 | 0.1995 | 1 | 0.3530 | 1 |
| L AMY → HTH | 0.2109 | 1 | -0.1282 | 1 | 0.4746 | 1 | 0.0000 | 0 |
| R AMY → VMPFC | -0.1938 | 1 | 0.1567 | 1 | -0.1634 | 1 | 0.0000 | 0 |
| R AMY → L VLPFC | 0.1993 | 1 | 0.0000 | 0 | -0.1915 | 1 | 0.2121 | 1 |
| R AMY → R VLPFC | 0.2310 | 1 | -0.1386 | 1 | -0.1084 | 1 | 0.0000 | 0 |
| R AMY → L AMY | 0.0714 | 0.774393937 | 0.0000 | 0 | -0.1607 | 1 | 0.3788 | 1 |
| R AMY → R AMY | 0.1800 | 1 | 0.0000 | 0 | 0.0000 | 0 | 0.1338 | 1 |
| R AMY → L FG | 0.3174 | 1 | -0.1954 | 1 | -0.1040 | 1 | 0.0000 | 0 |
| R AMY → R FG | 0.1033 | 1 | -0.1356 | 1 | -0.1775 | 1 | 0.0000 | 0 |
| R AMY → HTH | -0.1159 | 1 | 0.0000 | 0 | -0.1085 | 1 | 0.1728 | 1 |
| L FG → VMPFC | 0.1540 | 1 | 0.0883 | 1 | -0.2468 | 1 | -0.1090 | 1 |
| L FG → L VLPFC | -0.1771 | 1 | 0.0000 | 0 | 0.0953 | 1 | -0.1698 | 1 |
| L FG → R VLPFC | -0.0852 | 1 | -0.0315 | 0.450366502 | 0.2634 | 1 | 0.0000 | 0 |
| L FG → L AMY | 0.0000 | 0 | -0.1294 | 1 | 0.0000 | 0 | 0.0000 | 0 |
| L FG → R AMY | 0.2877 | 1 | 0.0511 | 0.603248969 | -0.0815 | 1 | 0.0000 | 0 |
| L FG → L FG | 0.2990 | 1 | 0.0000 | 0 | 0.1878 | 1 | 0.0000 | 0 |
| L FG → R FG | 0.0000 | 0 | -0.1299 | 1 | 0.0000 | 0 | -0.2052 | 1 |
| L FG → HTH | 0.1624 | 1 | 0.1638 | 1 | -0.1136 | 1 | 0.0000 | 0 |
| R FG → VMPFC | 0.0000 | 0 | -0.0650 | 0.750691828 | 0.2455 | 1 | 0.0000 | 0 |
| R FG → L VLPFC | 0.2600 | 1 | 0.1502 | 1 | 0.0000 | 0 | 0.0442 | 0.594831845 |
| R FG → R VLPFC | 0.1955 | 1 | 0.0667 | 1 | -0.1815 | 1 | 0.0000 | 0 |
| R FG → L AMY | 0.0000 | 0 | -0.1066 | 1 | 0.1599 | 1 | -0.1155 | 1 |
| R FG → R AMY | -0.1782 | 1 | -0.1744 | 1 | 0.1052 | 1 | 0.1038 | 1 |
| R FG → L FG | 0.2479 | 1 | 0.0000 | 0 | -0.0541 | 0.720143964 | -0.1358 | 1 |
| R FG → R FG | -0.1701 | 1 | 0.0000 | 0 | 0.0000 | 0 | 0.0000 | 0 |
| R FG → HTH | 0.0000 | 0 | 0.0000 | 0 | 0.0000 | 0 | -0.0555 | 0.708861013 |
| HTH → VMPFC | 0.0000 | 0 | 0.0902 | 1 | 0.0000 | 0 | -0.0699 | 1 |
| HTH → L VLPFC | -0.2063 | 1 | 0.0947 | 1 | -0.1203 | 1 | -0.1883 | 1 |
| HTH → R VLPFC | -0.2220 | 1 | 0.1923 | 1 | -0.2521 | 1 | -0.2514 | 1 |
| HTH → L AMY | -0.2153 | 1 | 0.0809 | 1 | -0.0976 | 1 | -0.1645 | 1 |
| HTH → R AMY | 0.0000 | 0 | 0.1748 | 1 | -0.1286 | 1 | 0.0000 | 0 |
| HTH → L FG | -0.3911 | 1 | 0.2700 | 1 | -0.1474 | 1 | 0.0000 | 0 |
| HTH → R FG | -0.3495 | 1 | 0.2637 | 1 | -0.2246 | 1 | -0.0816 | 1 |
| HTH → HTH | 0.0000 | 0 | 0.0000 | 0 | 0.2313 | 1 | -0.1912 | 1 |

**Supplemental Table S6.** Results of linear regression analyses showing associations between EC and cumulative alcohol use, anxiety, and depression scores in AUD group. For each node-to-node connection in each linear regression analysis, the regression coefficient (beta) reflects EC magnitude in Hz per unit of measure for alcohol usage, anxiety score, and depression score, respectively. The Bayesian posterior probabilities (PP) between 0 and 1 (0≤PP≤1) is shown for each calculation. For interpretation purposes: Larger magnitude ECs with Bayesian-PP ≥0.95 are considered reliable. Test-retest reliability of ECs with larger magnitudes is higher. Abbreviations: EC=effective connectivity, PP=posterior probability, L=left, and R=right. VMPFC = ventromedial prefrontal cortex, VLPFC = ventrolateral prefrontal cortex, AMY = amygdala, FG = fusform gyrus, HTH = hypothalamus.

|  | **Linear regression** between EC and cumulative alcohol use in AUD participants (n=70) | | **Linear regression** between EC and anxiety score in AUD participants (n=70) | | **Linear regression** between EC and depression score in AUD participants (n=70) | |
| --- | --- | --- | --- | --- | --- | --- |
| **Connectivity** | **beta** | **PP** | **beta** | **PP** | **beta** | **PP** |
| VMPFC → VMPFC | 0.0000 | 0 | 0.0000 | 0 | 0.0000 | 0 |
| VMPFC → L VLPFC | 0.0000 | 0 | 0.0000 | 0 | 0.0000 | 0 |
| VMPFC → R VLPFC | 0.0000 | 0 | 0.0000 | 0 | 0.0000 | 0 |
| VMPFC → L AMY | 0.0000 | 0 | 0.0000 | 0 | 0.0000 | 0 |
| VMPFC → R AMY | 0.0000 | 0 | 0.0000 | 0 | 0.0000 | 0 |
| VMPFC → L FG | 0.0000 | 0 | 0.0000 | 0 | 0.0000 | 0 |
| VMPFC → R FG | 0.0000 | 0 | 0.0000 | 0 | 0.0000 | 0 |
| VMPFC → HTH | 0.0000 | 0 | 0.0000 | 0 | 0.0000 | 0 |
| L VLPFC → VMPFC | 0.0000 | 0 | 0.0000 | 0 | 0.0000 | 0 |
| L VLPFC → L VLPFC | 0.0000 | 0 | 0.0000 | 0 | 0.0000 | 0 |
| L VLPFC → R VLPFC | 0.0000 | 0 | 0.0000 | 0 | 0.0000 | 0 |
| L VLPFC → L AMY | 0.0000 | 0 | 0.0000 | 0 | 0.0000 | 0 |
| L VLPFC → R AMY | 0.0000 | 0 | 0.0000 | 0 | 0.0000 | 0 |
| L VLPFC → L FG | 0.0000 | 0 | 0.0000 | 0 | 0.0000 | 0 |
| L VLPFC → R FG | 0.0000 | 0 | 0.0000 | 0 | 0.0000 | 0 |
| L VLPFC → HTH | 0.0000 | 0 | 0.0000 | 0 | 0.0000 | 0 |
| R VLPFC → VMPFC | 0.0000 | 0 | 0.0000 | 0 | 0.0000 | 0 |
| R VLPFC → L VLPFC | 0.0000 | 0 | 0.0000 | 0 | 0.0000 | 0 |
| R VLPFC → R VLPFC | 0.0000 | 0 | 0.0000 | 0 | 0.0000 | 0 |
| R VLPFC → L AMY | 0.0000 | 0 | 0.0000 | 0 | 0.0000 | 0 |
| R VLPFC → R AMY | 0.0000 | 0 | 0.0000 | 0 | 0.0000 | 0 |
| R VLPFC → L FG | 0.0000 | 0 | 0.0000 | 0 | 0.0000 | 0 |
| R VLPFC → R FG | 0.0000 | 0 | 0.0000 | 0 | 0.0000 | 0 |
| R VLPFC → HTH | 0.0000 | 0 | 0.0000 | 0 | 0.0000 | 0 |
| L AMY → VMPFC | 0.0000 | 0 | 0.0000 | 0 | 0.0000 | 0 |
| L AMY → L VLPFC | 0.0000 | 0 | 0.0000 | 0 | 0.0000 | 0 |
| L AMY → R VLPFC | 0.0000 | 0 | 0.0000 | 0 | 0.0000 | 0 |
| L AMY → L AMY | 0.0000 | 0 | 0.0000 | 0 | 0.0000 | 0 |
| L AMY → R AMY | 0.0000 | 0 | 0.0000 | 0 | 0.0000 | 0 |
| L AMY → L FG | 0.0000 | 0 | 0.0000 | 0 | 0.0000 | 0 |
| L AMY → R FG | 0.0000 | 0 | 0.0000 | 0 | 0.0000 | 0 |
| L AMY → HTH | 0.0000 | 0 | 0.0000 | 0 | 0.0000 | 0 |
| R AMY → VMPFC | 0.0000 | 0 | 0.0000 | 0 | 0.0000 | 0 |
| R AMY → L VLPFC | 0.0000 | 0 | 0.0000 | 0 | 0.0000 | 0 |
| R AMY → R VLPFC | 0.0000 | 0 | 0.0000 | 0 | 0.0000 | 0 |
| R AMY → L AMY | 0.0000 | 0 | 0.0000 | 0 | 0.0000 | 0 |
| R AMY → R AMY | 0.0000 | 0 | 0.0000 | 0 | 0.0000 | 0 |
| R AMY → L FG | 0.0000 | 0 | 0.0000 | 0 | –0.0221 | 0.9479 |
| R AMY → R FG | 0.0000 | 0 | 0.0000 | 0 | 0.0000 | 0 |
| R AMY → HTH | 0.0000 | 0 | 0.0000 | 0 | 0.0287 | 0.9858 |
| L FG → VMPFC | 0.0000 | 0 | 0.0000 | 0 | 0.0000 | 0 |
| L FG → L VLPFC | 0.0000 | 0 | 0.0000 | 0 | 0.0000 | 0 |
| L FG → R VLPFC | 0.0000 | 0 | 0.0000 | 0 | 0.0000 | 0 |
| L FG → L AMY | 0.0000 | 0 | 0.0000 | 0 | 0.0000 | 0 |
| L FG → R AMY | 0.0000 | 0 | 0.0000 | 0 | 0.0000 | 0 |
| L FG → L FG | 0.0000 | 0 | 0.0000 | 0 | 0.0000 | 0 |
| L FG → R FG | 0.0000 | 0 | 0.0000 | 0 | 0.0000 | 0 |
| L FG → HTH | 0.0000 | 0 | 0.0000 | 0 | 0.0000 | 0 |
| R FG → VMPFC | 0.0000 | 0 | 0.0000 | 0 | 0.0000 | 0 |
| R FG → L VLPFC | 0.0000 | 0 | 0.0000 | 0 | 0.0000 | 0 |
| R FG → R VLPFC | 0.0000 | 0 | 0.0000 | 0 | 0.0000 | 0 |
| R FG → L AMY | 0.0000 | 0 | 0.0000 | 0 | 0.0000 | 0 |
| R FG → R AMY | 0.0000 | 0 | 0.0000 | 0 | –0.0090 | 0.5248 |
| R FG → L FG | 0.0000 | 0 | 0.0000 | 0 | 0.0000 | 0 |
| R FG → R FG | 0.0000 | 0 | 0.0000 | 0 | 0.0000 | 0 |
| R FG → HTH | 0.0000 | 0 | 0.0000 | 0 | 0.0000 | 0 |
| HTH → VMPFC | 0.0000 | 0 | 0.0000 | 0 | 0.0000 | 0 |
| HTH → L VLPFC | 0.0000 | 0 | 0.0000 | 0 | 0.0000 | 0 |
| HTH → R VLPFC | 0.0000 | 0 | 0.0000 | 0 | 0.0000 | 0 |
| HTH → L AMY | 0.0000 | 0 | 0.0000 | 0 | 0.0000 | 0 |
| HTH → R AMY | 0.0000 | 0 | 0.0000 | 0 | 0.0000 | 0 |
| HTH → L FG | 0.0000 | 0 | 0.0000 | 0 | 0.0000 | 0 |
| HTH → R FG | 0.0000 | 0 | 0.0000 | 0 | 0.0000 | 0 |
| HTH → HTH | 0.0000 | 0 | 0.0000 | 0 | 0.0000 | 0 |

**Supplemental Table S7.** Results of linear regression analyses showing associations between EC and cumulative alcohol use, anxiety, and depression scores in CON group. For each node-to-node connection in each linear regression analysis, the regression coefficient (beta) reflects EC magnitude in Hz per unit of measure for alcohol usage, anxiety score, and depression score, respectively. The Bayesian posterior probabilities (PP) between 0 and 1 (0≤PP≤1) is shown for each calculation. For interpretation purposes: Larger magnitude ECs with Bayesian-PP ≥0.95 are considered reliable. Test-retest reliability of ECs with larger magnitudes is higher. Abbreviations: EC=effective connectivity, PP=posterior probability, L=left, and R=right. VMPFC = ventromedial prefrontal cortex, VLPFC = ventrolateral prefrontal cortex, AMY = amygdala, FG = fusform gyrus, HTH = hypothalamus.

|  | **Linear regression** between EC and cumulative alcohol use in CON participants (n=70) | | **Linear regression** between EC and anxiety score in CON participants (n=70) | | **Linear regression** between EC and depression score in CON participants (n=70) | |
| --- | --- | --- | --- | --- | --- | --- |
| **Connectivity** | **beta** | **PP** | **beta** | **PP** | **beta** | **PP** |
| VMPFC → VMPFC | 0.0000 | 0 | 0.0000 | 0 | 0.0000 | 0 |
| VMPFC → L VLPFC | 0.0000 | 0 | 0.0000 | 0 | 0.0000 | 0 |
| VMPFC → R VLPFC | 0.0000 | 0 | 0.0000 | 0 | 0.0000 | 0 |
| VMPFC → L AMY | 0.0000 | 0 | 0.0000 | 0 | 0.0000 | 0 |
| VMPFC → R AMY | 0.0000 | 0 | 0.0000 | 0 | 0.0000 | 0 |
| VMPFC → L FG | 0.0000 | 0 | 0.0000 | 0 | 0.0000 | 0 |
| VMPFC → R FG | 0.0000 | 0 | 0.0000 | 0 | 0.0000 | 0 |
| VMPFC → HTH | 0.0000 | 0 | 0.0000 | 0 | 0.0000 | 0 |
| L VLPFC → VMPFC | 0.0000 | 0 | 0.0000 | 0 | 0.0000 | 0 |
| L VLPFC → L VLPFC | 0.0000 | 0 | 0.0000 | 0 | 0.0000 | 0 |
| L VLPFC → R VLPFC | 0.0000 | 0 | 0.0000 | 0 | 0.0000 | 0 |
| L VLPFC → L AMY | 0.0000 | 0 | 0.0000 | 0 | 0.0000 | 0 |
| L VLPFC → R AMY | 0.0000 | 0 | 0.0000 | 0 | 0.0000 | 0 |
| L VLPFC → L FG | 0.0000 | 0 | 0.0000 | 0 | 0.0000 | 0 |
| L VLPFC → R FG | 0.0000 | 0 | 0.0000 | 0 | 0.0000 | 0 |
| L VLPFC → HTH | 0.0000 | 0 | 0.0000 | 0 | 0.0000 | 0 |
| R VLPFC → VMPFC | 0.0000 | 0 | 0.0000 | 0 | 0.0000 | 0 |
| R VLPFC → L VLPFC | 0.0000 | 0 | 0.0000 | 0 | 0.0000 | 0 |
| R VLPFC → R VLPFC | 0.0000 | 0 | 0.0000 | 0 | 0.0000 | 0 |
| R VLPFC → L AMY | 0.0000 | 0 | 0.0000 | 0 | 0.0000 | 0 |
| R VLPFC → R AMY | 0.0000 | 0 | 0.0000 | 0 | 0.0000 | 0 |
| R VLPFC → L FG | 0.0000 | 0 | 0.0000 | 0 | 0.0000 | 0 |
| R VLPFC → R FG | 0.0000 | 0 | 0.0000 | 0 | 0.0000 | 0 |
| R VLPFC → HTH | 0.0000 | 0 | 0.0000 | 0 | 0.0000 | 0 |
| L AMY → VMPFC | 0.0000 | 0 | 0.0000 | 0 | 0.0000 | 0 |
| L AMY → L VLPFC | 0.0000 | 0 | 0.0000 | 0 | 0.0000 | 0 |
| L AMY → R VLPFC | 0.0000 | 0 | 0.0000 | 0 | 0.0000 | 0 |
| L AMY → L AMY | 0.0000 | 0 | 0.0000 | 0 | 0.0000 | 0 |
| L AMY → R AMY | 0.0000 | 0 | 0.0000 | 0 | 0.0000 | 0 |
| L AMY → L FG | 0.0000 | 0 | 0.0000 | 0 | 0.0000 | 0 |
| L AMY → R FG | 0.0000 | 0 | 0.0000 | 0 | 0.0000 | 0 |
| L AMY → HTH | 0.0000 | 0 | 0.0000 | 0 | 0.0000 | 0 |
| R AMY → VMPFC | 0.0000 | 0 | 0.0000 | 0 | 0.0000 | 0 |
| R AMY → L VLPFC | 0.0000 | 0 | 0.0000 | 0 | 0.0000 | 0 |
| R AMY → R VLPFC | 0.0000 | 0 | 0.0000 | 0 | 0.0000 | 0 |
| R AMY → L AMY | 0.0000 | 0 | 0.0000 | 0 | 0.0000 | 0 |
| R AMY → R AMY | 0.0000 | 0 | 0.0000 | 0 | 0.0000 | 0 |
| R AMY → L FG | 0.0000 | 0 | 0.0000 | 0 | 0.0000 | 0 |
| R AMY → R FG | 0.0000 | 0 | 0.0000 | 0 | 0.0000 | 0 |
| R AMY → HTH | 0.0000 | 0 | 0.0000 | 0 | 0.0000 | 0 |
| L FG → VMPFC | 0.0000 | 0 | 0.0000 | 0 | 0.0000 | 0 |
| L FG → L VLPFC | 0.0000 | 0 | 0.0000 | 0 | 0.0000 | 0 |
| L FG → R VLPFC | 0.0000 | 0 | 0.0000 | 0 | 0.0000 | 0 |
| L FG → L AMY | 0.0000 | 0 | 0.0000 | 0 | 0.0000 | 0 |
| L FG → R AMY | 0.0000 | 0 | 0.0000 | 0 | 0.0000 | 0 |
| L FG → L FG | 0.0000 | 0 | 0.0000 | 0 | 0.0000 | 0 |
| L FG → R FG | 0.0000 | 0 | 0.0000 | 0 | 0.0000 | 0 |
| L FG → HTH | 0.0000 | 0 | 0.0000 | 0 | 0.0000 | 0 |
| R FG → VMPFC | 0.0000 | 0 | 0.0000 | 0 | 0.0000 | 0 |
| R FG → L VLPFC | 0.0000 | 0 | 0.0000 | 0 | 0.0000 | 0 |
| R FG → R VLPFC | 0.0000 | 0 | 0.0000 | 0 | 0.0000 | 0 |
| R FG → L AMY | 0.0000 | 0 | 0.0000 | 0 | 0.0000 | 0 |
| R FG → R AMY | 0.0000 | 0 | 0.0000 | 0 | 0.0000 | 0 |
| R FG → L FG | 0.0000 | 0 | 0.0000 | 0 | 0.0000 | 0 |
| R FG → R FG | 0.0000 | 0 | 0.0000 | 0 | 0.0000 | 0 |

**References**

Friston, K.J., Harrison, L., Penny, W., 2003. Dynamic causal modelling. Neuroimage 19, 1273-1302. PMID: 12948688.

Friston, K.J., Penny, W., 2003. Posterior probability maps and SPMs. Neuroimage 19, 1240-1249.

Orr, J.M., Paschall, C.J., Banich, M.T., 2016. Recreational marijuana use impacts white matter integrity and subcortical (but not cortical) morphometry. Neuroimage Clin 12, 47-56.

Van Overwalle, F., Van de Steen, F., Marien, P., 2019. Dynamic causal modeling of the effective connectivity between the cerebrum and cerebellum in social mentalizing across five studies. Cogn Affect Behav Neurosci 19, 211-223.

Xia, M., Wang, J., He, Y., 2013. BrainNet Viewer: a network visualization tool for human brain connectomics. PLoS ONE 8, e68910.
